# Molecular phylogeny of *Planaltina* Böhlke (Characidae: Stevardiinae) and comments on the definition and geographic distribution of the genus, with description of a new species

**DOI:** 10.1371/journal.pone.0196291

**Published:** 2018-05-16

**Authors:** Gabriel de Carvalho Deprá, Weferson Júnio da Graça, Carla Simone Pavanelli, Gleisy Semencio Avelino, Claudio Oliveira

**Affiliations:** 1 Programa de Pós-Graduação em Ecologia de Ambientes Aquáticos Continentais, Universidade Estadual de Maringá, Maringá, Paraná, Brazil; 2 Departamento de Biologia, Universidade Estadual de Maringá, Maringá, Paraná, Brazil; 3 Núcleo de Pesquisas em Limnologia, Ictiologia e Aquicultura, Universidade Estadual de Maringá, Maringá, Paraná, Brazil; 4 Instituto de Biociências, Departamento de Morfologia, Universidade Estadual Paulista Júlio de Mesquita Filho, Botucatu, São Paulo, Brazil; National Cheng Kung University, TAIWAN

## Abstract

A molecular phylogeny of *Planaltina*, including the three previously described species and an undescribed species, is presented. The monophyly of the genus, included in Diapomini, is strongly supported. Its sister group, the remaining Diapomini, includes only species without modified caudal-fin squamation in the present analysis (species of *Diapoma* with caudal organs were not sampled). *Creagrutus* is sister to *Planaltina* plus remaining Diapomini instead of *Planaltina* being sister to *Creagrutus* plus Diapomini, as a previous analysis had suggested. Species of *Planaltina* form two clades: *P*. *britskii* plus the new species, with low support (< 50%); and *P*. *myersi* plus *P*. *glandipedis*, with higher support. *Planaltina* is rediagnosed from all Characidae based on the morphology of the caudal organ, the absence of a humeral spot and the presence of a complete lateral line. Comments on the caudal-fin squamation of *Diapoma* and *Lepidocharax burnsi*, on the type-series of *L*. *burnsi* and on the geographic distribution of *Planaltina* and *Lepidocharax* species are provided. Finally, a formal description of the aforementioned new species and a novel identification key to *Planaltina* are presented.

## Introduction

The past ten years brought several advancements to the understanding of the characid phylogeny. Much work was directed to the Stevardiinae (as defined by Thomaz *et al*. [1]), with highly diverging results. Data sources included the traditional external morphology and osteology [2, 3], but also histology [4, 5] and DNA [1, 6, 7]. Available phenotypic studies [5, 8] seem to have overestimated resemblance between caudal organs in the former members of the Glandulocaudinae (*sensu* Weitzman & Menezes [8]), masking their now-evident polyphyly [1, 7]. That is shown by late molecular studies, which also deeply rearranged the classification of the group, sinking a few “specialized” genera and giving new combinations for species previously placed in large, “catch-all” genera [1]. The apparently homoplastic evolution of caudal organs, specialized teeth etc. in stevardiine fishes sheds doubt on the monophyletic origin of some poorly defined genera.

Böhlke [9] described *Planaltina* and its type-species *P*. *myersi* Böhlke based on a single male specimen, and diagnosed it from all other Characidae by an extensive combination of characters, mostly meristic. Weitzman & Menezes [8] observed that in *Planaltina* males and females have externally indistinguishable caudal organs, a character shared only with *Acrobrycon* Eigenmann & Pearson and *Diapoma* Cope within Stevardiinae, interpreting it as a synapomorphy for those genera and placing them in a redefined tribe Diapomini (posteriorly found to be polyphyletic [1, 7]). Subsequently, Menezes *et al*. [10] redefined *Planaltina* simply by diagnosing it from *Acrobrycon* and *Diapoma*, based mainly in the presence of 1–2 scales in the dorsal border of the caudal pouch opening (*vs*. 4–7 and 3–6, respectively; posteriorly, Menezes & Weitzman [11] and Arcila *et al*. [12] recorded 4–8 scales in both genera) and the nearly spherical sperm nuclei. Nevertheless, it is possible to observe in Menezes *et al*. ([10], Figs. 28, 33) that *P*. *glandipedis* Menezes, Weitzman & Burns occasionally exhibits more than two scales in the dorsal border of the caudal pouch opening and *P*. *britskii* Menezes, Weitzman & Burns is sexually dimorphic for the shape of the caudal-fin scales, which means some changes in the definition of *Planaltina* are needed.

The known geographic distribution of *Planaltina* presently includes only those localities recorded by Menezes *et al*. [10], Graça & Pavanelli [13] and Araújo & Tejerina-Garro [14]: *P*. *britskii* has been reported from the Grande, São José dos Dourados and Tietê river basins, as well as from other, smaller tributaries of the rio Paraná in the State of São Paulo and from the upper rio Paraná floodplain; *P*. *glandipedis*, from the portion of the rio Tietê basin draining the cuestas of the State of São Paulo; and *P*. *myersi*, from the Corumbá and Ouvidor river basins, both emptying in the rio Paranaíba. A recently discovered species (*Planaltina* sp. of Frota *et al*. [15]) from the Ivaí and Piquiri river basins, south to the known geographic range of *Planaltina*, agrees only partially with the diagnosis of the genus by Menezes *et al*. [10]; as in *P*. *glandipedis*, some individuals of the new species present as much as four scales in the dorsal border of the caudal pouch.

This paper aims to investigate the relationships of *Planaltina* based on a molecular phylogenetic analysis, including the new species, in order to test its monophyly, hence permitting the proposition of a revised diagnosis of the genus (with comments on the caudal-fin squamation in *Diapoma* and *Lepidocharax*). It also expands the known geographic range of all previously described *Planaltina* species. Additionally, we provide a formal description of the aforementioned new species, a novel identification key to the species of *Planaltina* and comments on the type-series of *L*. *burnsi*.

## Material and methods

### Molecular data collection

Total DNA was extracted from ethanol-preserved muscle samples using the DNeasy Tissue Extraction Kit (Qiagen) following the manufacturer’s instructions. Partial sequences of the mitochondrial genes 16SrRNA and Cytochrome *b* (CytB) and the nuclear genes recombination activating gene 1 (Rag1), recombination activating gene 2 (Rag2) and myosin heavy chain 6 cardiac muscle alpha (Myh6) were amplified by polymerase chain reaction (PCR) with the primers listed in S1 File. Amplifications were performed in a total volume of 25 μl consisting of 2.5 μl 10X buffer (10 mM Tris-HCL, 15 mM MgCl_2_), 0.5 μl MgCl_2_ (50 mM), 0.5 μl each primer (5 μM); 0.4 μl of dNTPs (200 nM of each), 0.2 μl Taq Platinum polymerase (Invitrogen; 5 U/μl), 1 μl template DNA (10–50 ng) and 19.4 μl ddH_2_O. The thermocycler profile used for the fragments 16SrRNA and CytB consisted of 35 cycles of 30 s at 95°C, 45–120 s at 50–55°C, and 90 s at 72°C. Nested PCR was used to amplify the nuclear genes Rag1, Rag2 and Myh6. Amplification conditions for these genes in both rounds of PCR consisted of 15 cycles of 30 s at 95°C, 45 s at 56°C (according to primer), and 30 s at 72°C followed by 15 cycles of 30 s at 95°C, 45 s at 54°C (according to primer), and 90 s at 72°C. PCR products were purified using ExoSap-IT® (USB Corporation), sequenced using the Big DyeTM Terminator v 3.1 Cycle Sequencing Ready Reaction Kit (Applied Biosystems), purified again by ethanol precipitation and loaded into an automatic sequencer 3130 Genetic Analyzer (Applied Biosystems) at Instituto de Biociências, Universidade Estadual Paulista, Botucatu, São Paulo, Brazil. Contigs were assembled and edited in Geneious Pro 8.1.8 [16]. In cases of unclear nucleotide identity, IUPAC ambiguity codes were applied. All obtained sequences were deposited in GenBank.

### Alignment and phylogenetic analysis

Sequences of each gene were aligned in Geneious Pro 8.1.8 [16] using the MUSCLE algorithm under default parameters, and the alignments were inspected by eye for any obvious misalignments that were subsequently corrected. Genetic distances among sequences were calculated in Mega 6 [17]. We estimated the index of substitution saturation (Iss) in DAMBE 5.2.31 [18] as described in Xia *et al*. [19, 20].

A set of six partitioning schemes ranging from 1 to 13 partitions was tested following the procedures outlined by Li *et al*. [21] using the AICc (Akaike Information Criterion, corrected for finite sample sizes). The best-fit model of nucleotide substitution was searched in Mega 6 [17] under default parameters using the AICc (see Posada & Buckley [22] for justification).

RAxML [23] running in the web servers RAxML-HPC2 on TG [24; 25] was used for all maximum likelihood analyses with a mixed partition model. Random starting trees were run for each independent ML tree search, and all other parameters were set to default values. All ML analyses were conducted following the 13 partitions scheme as suggested by the AICc. Topological robustness was investigated using 1,000 non-parametric bootstrap replicates.

The ingroup was composed of the four species of *Planaltina*: *P*. *britskii* (specimen 17243 –LBP 2598 Rio Paraná Basin, 21°00'46.6''S 49°41'25.1''W, Miraluz, São Paulo), *P*. *glandipedis* (specimen 61094 –LPB 14618, Rio Paraná Basin, 22°44'50.2''S 48°28'30.5''W, Botucatu, São Paulo), *P*. *myersi* (specimen 75276 –LBP 11680, Rio Paraná Basin, 17°56'39.5''S 46°58'09.6''W, Claro de Minas, Minas Gerais), and the new species described herein (specimens 75269, 75270 –LBP 18902, Rio Paraná Basin, rio Maria Flora, 24°36'32.0"S 51°15'31.0"W, Cândido de Abreu, Paraná). To test the position of the species of *Planaltina* in Characidae we use as outgroup species belonging to the families Chalceidae, Triportheidae, Gasteropelecidae, Bryconidae, Acestrorhynchidae, Iguanodectidae, and Characidae. Molecular data for these families were produced by Oliveira *et al*. [7], Tagliacollo *et al*. [26], Mariguela *et al*. [27], and Ferreira *et al*. [28]. All specimens for this study were collected in accordance with Brazilian laws under a permanent scientific collection license in the name of CO (IBAMA-SISBIO, 13843–1). Additionally, this survey was carried out in strict accordance with the recommendations from the National Council for the Control of Animal Experimentation and the Federal Board of Veterinary Medicine. The studied material was deposited in the Laboratório de Biologia e Genética de Peixes (LBP), Instituto de Biociências, Universidade Estadual Paulista, Botucatu, São Paulo, Brazil. The fish collections were authorized by Instituto Chico Mendes de Conservação da Biodiversidade (ICMBio) to WJG (Sisbio license #14028–1) and the research was conducted in accordance with the policies of the Ethical Conduct Committee on Animal Use (CEUA # 002/2012 and # 5680160117, which authorize the project "Morfologia, moléculas e biogeografia da ictiofauna de rios e riachos de diferentes bacias hidrográficas paranaenses", which led to the discovery of the new species described herein) as administered by the Universidade Estadual de Maringá, Maringá, Brazil. The fishes captured were anesthetized and euthanized with an overdose of benzocaine following Brazilian guide to good practice for euthanasia in animals by Federal Council of Veterinary Medicine, available at: http://portal.cfmv.gov.br/uploads/files/Guia%20de%20Boas%20Pr%C3%A1ticas%20para%20Eutanasia.pdf.pdf.

### Morphological data

Counts and measurements are the same as in Menezes *et al*. [10]. Principal caudal-fin rays are numbered from the longest dorsal unbranched ray (1) to the longest ventral unbranched ray (19). Count values were taken mostly from the left side of the specimens, but information about right-side counts is given when pertinent. Meristic data marked by an asterisk are the values for holotype. Numbers in parentheses after a count value represent the frequency of occurrence in the sample. Lots with measured specimens are marked with an asterisk. Diagnostic characters were examined in all specimens. The numbers of caudal-fin procurrent rays and posterior dentary teeth were annotated only for cleared and stained specimens and for entire alcohol-preserved specimens in which a precise count was possible. Sex was determined by the presence or absence of hooks on anal-fin rays. Specimens smaller than the minimum-sized hook-bearing specimens of each species were considered as young; above that size, specimens not bearing hooks were treated as females. Lots excluded from the type-series are poorly preserved and have important structures damaged. Complete geographic references are provided for all specimens of *Planaltina* and *Lepidocharax*, but not for remaining comparative specimens. Elevations of collection sites were estimated in the Google® Earth® version 7.1.5.1557 software.

### Collection acronyms

DZSJRP, Departamento de Zoologia e Botânica da Universidade Estadual Paulista “Júlio de Mesquita Filho”, São José do Rio Preto; LBP, Laboratório de Biologia e Genética de Peixes, Universidade Estadual Paulista “Júlio de Mesquita Filho”, Botucatu; MCP, Museu de Ciências e Tecnologia, Pontifícia Universidade Católica do Rio Grande do Sul, Porto Alegre; NUP, Coleção Ictiológica do Núcleo de Pesquisas em Limnologia, Ictiologia e Aquicultura, Universidade Estadual de Maringá, Maringá; MZUSP, Museu de Zoologia da Universidade de São Paulo, São Paulo; UFRGS, Departamento de Zoologia, Instituto de Biociências, Universidade Federal do Rio Grande do Sul, Porto Alegre.

### Nomenclatural acts

The electronic edition of this article conforms to the requirements of the amended International Code of Zoological Nomenclature, and hence the new names contained herein are available under that Code from the electronic edition of this article. This published work and the nomenclatural acts it contains have been registered in ZooBank, the online registration system for the ICZN. The ZooBank LSIDs (Life Science Identifiers) can be resolved and the associated information viewed through any standard web browser by appending the LSID to the prefix “http://zoobank.org/”. The LSID for this publication is: urn:lsid:zoobank.org:pub:573B0705-D1F7-4C26-8E56-BD2F2D18E7B6. The electronic edition of this work was published in a journal with an ISSN, and has been archived and is available from the following digital repositories: PubMed Central, LOCKSS.

## Results

### Relationships of *Planaltina*

**Fig 1:**
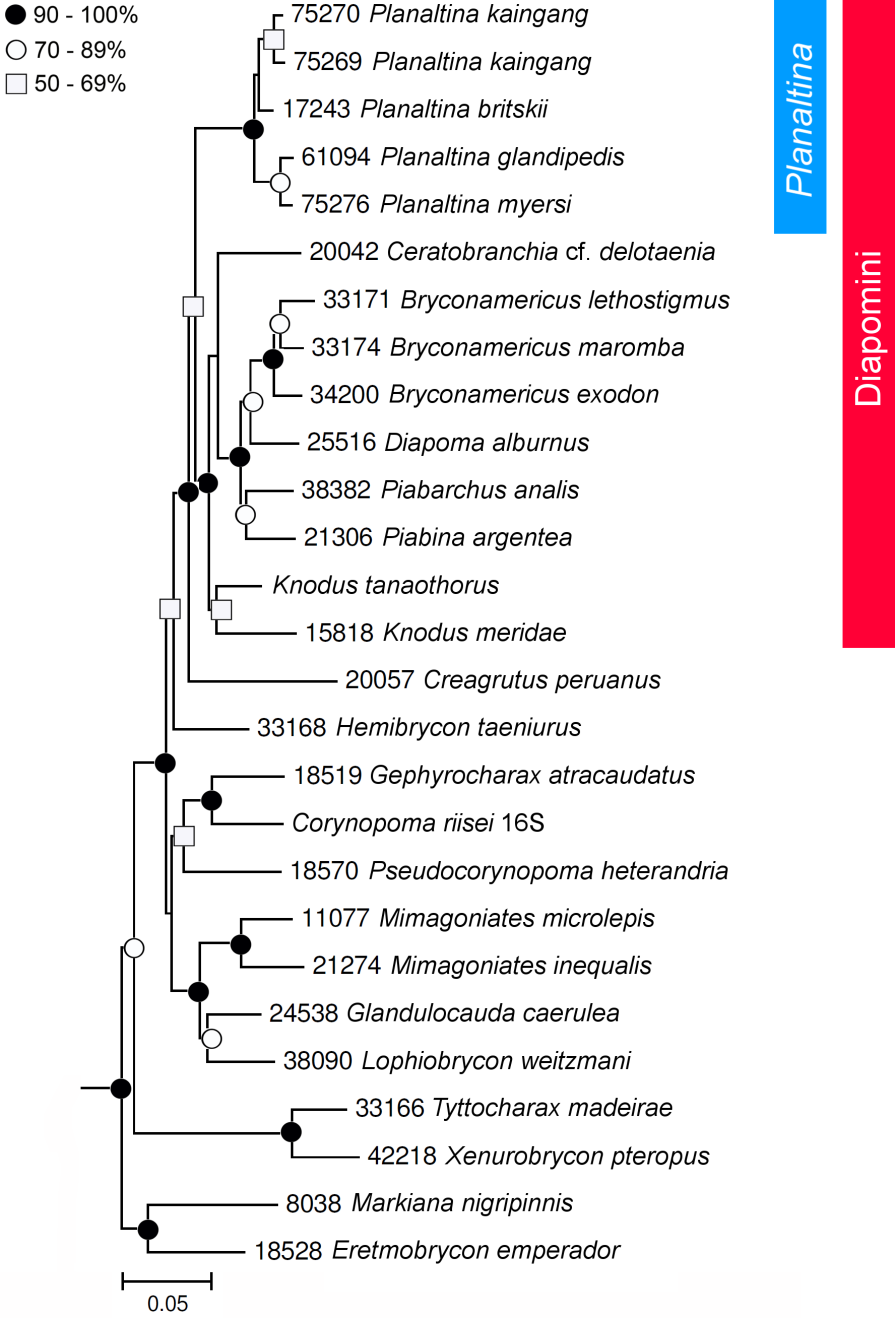
Best maximum likelihood tree of the Stevardiinae obtained in the partitioned analysis of the concatenated dataset showing the relationships among *Planaltina* species. Complete tree is in S1 Fig.

*Planaltina* was recovered within Diapomini, as sister to all other taxa included in the tribe (Fig 1). Of these, all, except some non-analyzed species of *Diapoma*, lack modified caudal-fin squamation. This contrasts with the results by Oliveira *et al*. [7], in which *Planaltina* is sister to *Creagrutus* plus Diapomini.

The monophyly of *Planaltina* is strongly supported, although its internal relationships were resolved with lower confidence. The new species was found as sister to *P*. *britskii*; that is the clade with the lowest support (< 50%). The low support (50–69%) of the clade including the two specimens of the new species (which were collected together) is probably due to the incompleteness of some sequences from those individuals and to the conservativeness of the molecular markers employed in the study, which present a relatively low divergence among *Planaltina* species. The other two species, *P*. *glandipedis* and *P*. *myersi*, clustered together, forming a well-supported clade (90–100%).

### Definition of *Planaltina*

**Fig 2:**
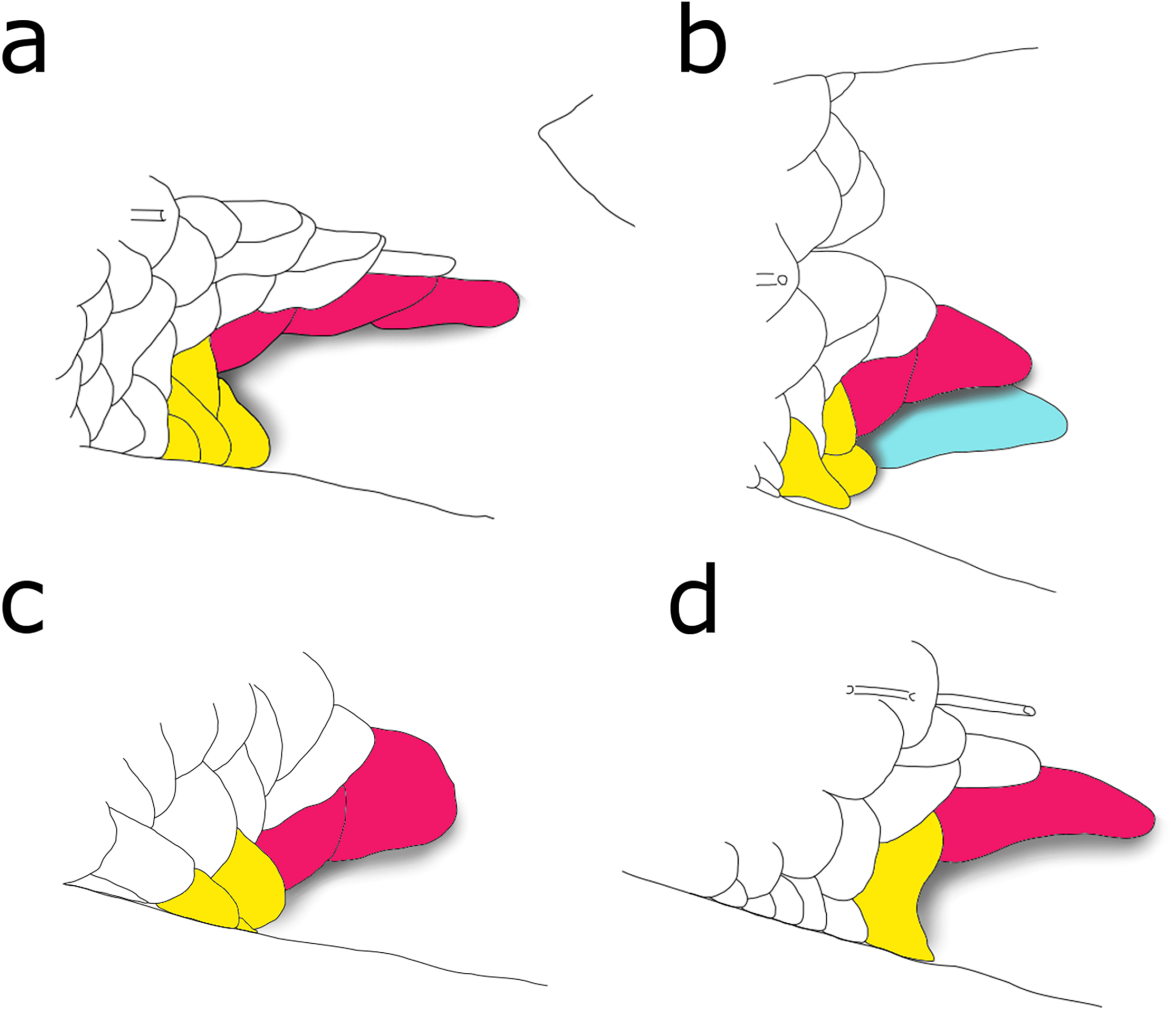
Schematic representation of the caudal-fin squamation of (a) the new species of *Planaltina* described herein; (b) male *P*. *britskii*; (c) female *P*. *britskii*; (d) *P*. *myersi*. Respectively, (b), (c) and (d) were adapted from figures 33.A, 33.B and 19 of Menezes *et al*. [10]. Pink-coloured scales are those forming the dorsal rim of the caudal-pouch opening; yellow-coloured scales are those forming the anterior border of the caudal pouch opening; the blue-coloured one is the adnate scale located in the inner side of the caudal pouch of male *P*. *britskii*. Shaded areas indicate the opening of the caudal pouch. Notice, in (c), that there are two scales forming the dorsal rim of the caudal-pouch opening, which is a rare condition in female *P*. *britskii* (only one scale usually present). The shape of these scales is, however, typical (that is, not particularly elongate as in male *P*. *britskii*).

*Planaltina* is diagnosed from all other Characidae by the following combination of external morphological characters: caudal pouch a single cavity completely restricted to the ventral caudal-fin lobe (*vs*. caudal pouch absent in most Characidae; occupying the ventral portion of the dorsal lobe in Glandulocaudini), its opening in a posteroventral position, never coriaceous or tubular (*vs*. opening posteriorly directed, pouch enclosed by skin ventrally in *Pseudocorynopoma*; tubular in *Chrysobrycon*, *Hysteronotus*); dorsal portion of caudal pouch formed by elongate, enlarged scales, which are attached to the fin by their long dorsal borders, and are never folded around themselves or ornamented with processes (*vs*. pouch scale deeper than long, with concave posterior margin in *Corynopoma* and *Gephyrocharax*; pouch scales not much elongate in *Phenacobrycon*; pouch scale ornamented with processes in *Hysteronotus*, *Pseudocorynopoma* and some Xenurobryconini); caudal pouch not including pored scales, never formed by a single enlarged scale (*vs*. including pored scales in *Argopleura* and *Landonia*; formed by single enlarged scale in all Xenurobryconini; see Fig 2, painted scales); humeral spot absent (*vs*. present in *Acrobrycon* and in species of *Diapoma* with modified caudal-fin squamation); lateral line complete (*vs*. incomplete in species of *Diapoma* with modified caudal-fin squamation);.

The following characters are also useful to distinguish *Planaltina* from other Stevardiinae: origin of dorsal fin slightly anterior to origin of anal fin (*vs*. origin of dorsal fin below base of last dorsal-fin ray in most stevardiines; anterior to origin of anal fin in several other genera, such as *Hysteronotus* and *Pterobrycon*); ventral region narrow but not keeled (*vs*. keeled in, *e*.*g*., *Pseudocorynopoma*); contact organs (hooks) never present posteriorly to the first ten branched anal-fin rays (*vs*. present in the first ten and in the last ten rays in *Phenacobrycon*); absence of modified caudal-fin rays (*vs*. modified rays present in, *e*.*g*., Glandulocaudini and Xenurobryconini); pelvic-fin rays usually i,5,i (rarely i,6; *vs*. commonly i,6 or i,7 in, *e*.*g*., *Bryconamericus* and *Knodus*). Male *P*. *britskii* can be distinguished from all other Characidae by the dorsal border of the caudal pouch opening formed by two elongate, enlarged scales; and by the presence, in the lower caudal-fin lobe, of an adnate scale (partially covered by the border of the pouch), whose posteroventral margin is bent laterad. Species of *Planaltina*, except *P*. *britskii*, are distinguished from all other Characidae, except *Acrobrycon* and *Diapoma*, by the equal development of the caudal pouch in both sexes (at least regarding external morphology). All species of *Planaltina* can be distinguished from *Acrobrycon* and species of *Diapoma* bearing modified caudal-fin scales by the absence of humeral spot; from *Acrobrycon*, by having few teeth in the maxilla (*vs*. teeth present along most of anterior margin of maxilla); and from *Diapoma* bearing modified caudal-fin scales, additionally by having complete lateral line (*vs*. incomplete).

**Fig 3:**
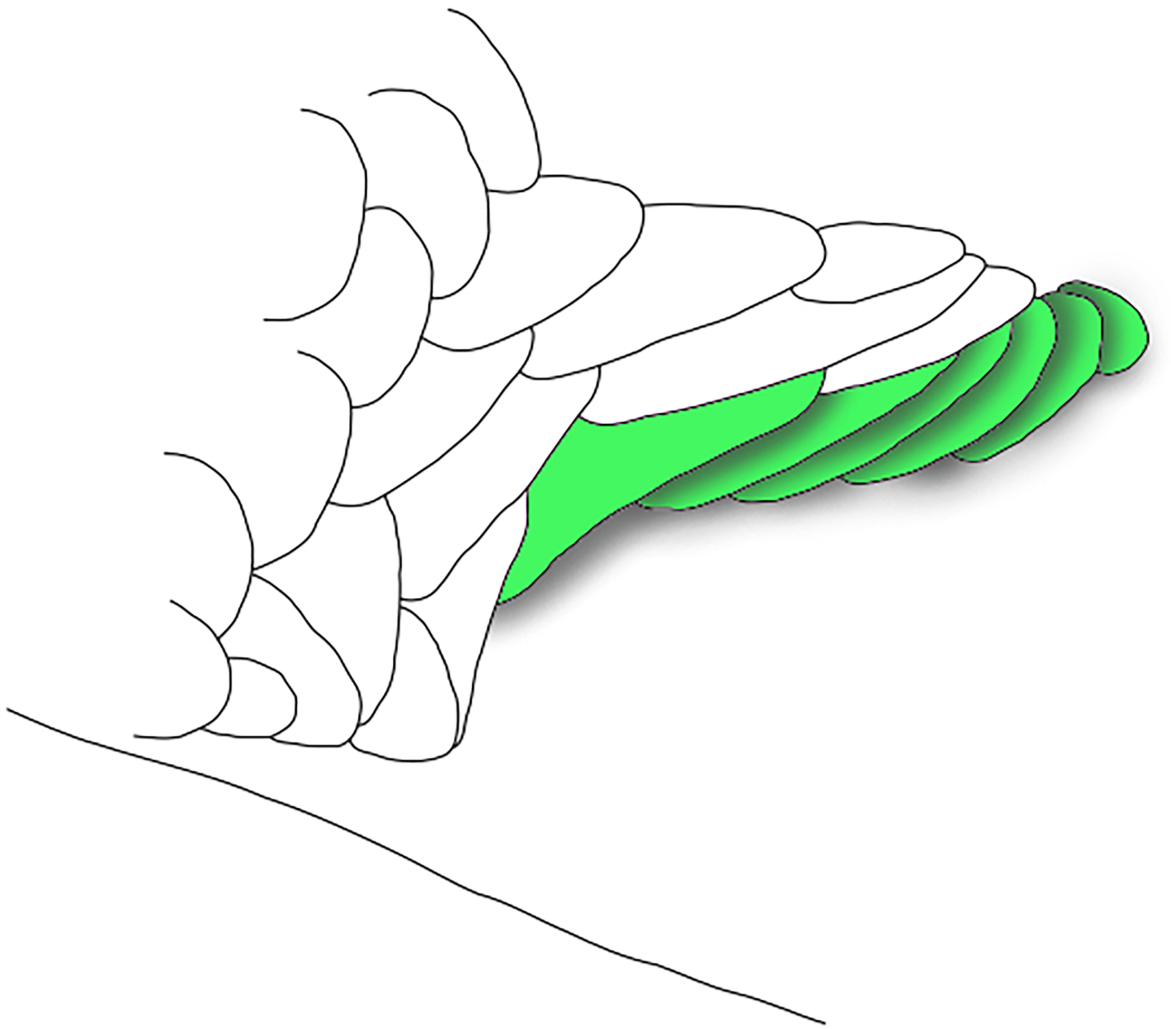
Schematic representation of the caudal-fin squamation of *Diapoma speculiferum*, adapted from Menezes & Weitzman ([11], fig. 30). Green-coloured scales are those forming the dorsal rim of the caudal-pouch opening. Shaded areas show that the scales are elevated, not adnate to the caudal-fin rays distally and not forming a single, well defined caudal pouch.

The amount of scales forming the dorsal border of the caudal pouch opening in *Planaltina* is 1–4, slightly overlapping the range of *Acrobrycon* and *Diapoma*, 4–8 [11, 12]. However, when more than one scale is present, they are imbricate and form a continuous wall that covers the pouch cavity. In contrast, in specimens of *D*. *speculiferum* Cope (type-species) and *D*. *terofali* Géry examined herein the largest, anteriormost “pouch scale” forms a cavity by itself and the other scales are partly included in this “pouch” (Fig 3). Each of the posterior scales has its posteroventral margin somewhat bent laterad, forming a smaller cavity underneath it, which could be considered as a smaller “pouch”. In *Planaltina*, only the adnate scale present on the inner side of the pouch of male *P*. *britskii* has its posteroventral margin bent laterad. However, it is located in a different position (Fig 2B), and is absent in females (Fig 2C). No sexual dimorphism was observed in the caudal-fin squamation of *D*. *speculiferum* and *D*. *terofali*.

Despite the caudal-fin squamation, the genus that most closely resembles *Planaltina* is *Lepidocharax*, especially *L*. *burnsi* Ferreira, Menezes & Quagio-Grassioto, whose body and mouth shape, dentition, meristics, morphometry and coloration completely overlap those of *Planaltina* species. The only external difference between those taxa is the presence of exclusively non-modified scales on the caudal fin in *L*. *burnsi*, hence the common misidentifications of this species in collections. Although Ferreira *et al*. [5] described the caudal-fin scales in both species of *Lepidocharax* as adnate, the scales in *L*. *burnsi* are not completely attached to the caudal-fin rays or to the thicker skin that covers the muscles inserted on their bases. Instead, in all specimens of *L*. *burnsi* examined herein (including the holotype and 127 paratypes) the caudal-fin scales are attached to the skin only by their anterior margins, being otherwise free from it. Thus, *L*. *burnsi* differs from all *Planaltina* species by lacking, in the ventral caudal-fin lobe, scales attached to the skin by their dorsal margins; however, truly adnate scales are not present (in comparison, they are found, for instance, in some *Knodus moenkhausii* (Eigenmann & Kennedy) specimens (NUP 6522), located more distally on caudal fin, attaching directly to the portion of the rays that is not covered by thick skin).

### Geographic distribution of *Planaltina*

**Fig 4:**
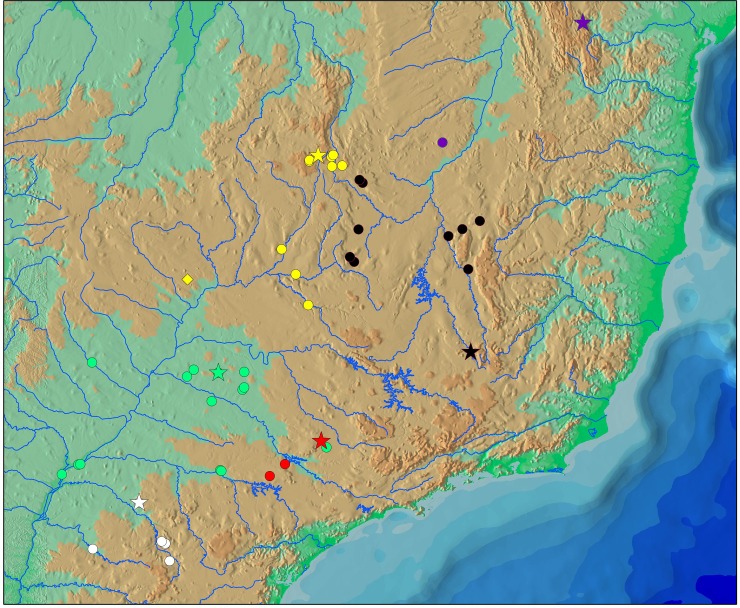
Map of the geographic distribution of *Planaltina* and *Lepidocharax* species, based mainly on specimens analyzed herein. White signs represent the new species of *Planaltina* described herein; green signs, *P*. *britskii*; red signs, *P*. *glandipedis*; yellow signs, *P*. *myersi*; black signs, *L*. *burnsi*; purple signs, *L*. *diamantina*. Stars represent type-localities; circles represent localities with specimens analyzed herein; and the lozenge-shaped mark represents the locality of one lot of *P*. *myersi* that was not analyzed herein, but whose identification was confirmed. Notice that a few previously listed localities [5, 10] are not shown herein.

Species of *Planaltina* are found in all major sub-basins of the upper rio Paraná biogeographic region, namely the Paranaíba, Grande, Tietê, Paranapanema, Ivaí and Piquiri river basins, as well as in the rio Paraná proper and some minor tributaries in both right and left banks (Fig 4). Additionally, *P*. *myersi* is found in the headwaters of the rio São Francisco basin close to the headwaters of the rio Corumbá.

In contrast to previous distributional data on *Planaltina* species [10], our data clearly show three cases of discontinuous geographic ranges: *P*. *glandipedis*, *P*. *myersi* and the new species. Of these, *P*. *myersi* occurs in the upper Paraná and São Francisco river basins. Isolated populations of *P*. *myersi* are very close geographically and morphologically indistinguishable. In that region, the headwaters of the Corumbá (upper rio Paraná basin) and Preto (rio São Francisco basin) river basins lay only a few hundred meters from each other, and the possibility of contact between them in the present should not be discarded. *Planaltina glandipedis* and the new species also occur each in two separate basins. Populations of *P*. *glandipedis* from the Tietê and Paranapanema river basins are situated near to one another, occurring in waterbodies draining the cuestas of the State of São Paulo. In contrast to *Planaltina glandipedis*, the new species is known from rather distant localities in the Ivaí and Piquiri river basins. Although Frota *et al*. ([15]; Fig 1) and Cavalli *et al*. (in press; Fig 1) have densely sampled both drainages, no *Planaltina* specimen was captured in their lower stretches, suggesting some sort of unknown ecological barrier. *Planaltina britskii* has a much wider geographic range, being notoriously allopatric to the new species and *P*. *myersi*. In fact, the only case of co-occurrence between two *Planaltina* species is that of *P*. *britskii* and *P*. *glandipedis* in the rio Tietê basin.

Carl Ternetz collected the holotype of *P*. *myersi* in the “Sarandi brook, Planaltina, Goyaz, Brazil” on September 21, 1923, but failed to determine whether this stream was a tributary to the upper Paraná or upper Tocantins river basin ([9], p. 267). Menezes *et al*. ([10], p. 564) ruled out the latter based on the fact that all specimens available to them originated from the upper rio Paraná basin, but provided no further information on the type-locality itself. Based on maps of the region [29, 30, 31], both earlier and posterior to the construction of the Brazilian Federal District (Brasília), we were able to delimit the type-locality of *Planaltina myersi* as “córrego Sarandi (tributary to the ribeirão Mestre-d’Armas, tributary to the rio São Bartolomeu, tributary to the rio Corumbá, tributary to the rio Paranaíba), upper rio Paraná basin, Brasília, Distrito Federal, approximately between 15°35’S 47°41’W and 15°35’S 47°44’W”. This locality is situated in Planaltina, an administrative region of Brasília, not in the northern portion of the former Planaltina municipality, which remains part of the State of Goiás and is almost completely drained by the rio Maranhão and its tributaries (rio Tocantins basin).

Concerning the presence of *Planaltina myersi* in the rio São Francisco basin, it had already been reported by Géry ([32], p. 358, key). Nevertheless, Géry’s [32] book lacks a list of examined material, thus it is possible that he has confused *Planaltina myersi* with *Lepidocharax burnsi*, which does not occur in sympatry with any *Planaltina* species, but is much more widely distributed in the rio São Francisco basin (Fig 4). Since no description of the caudal-fin squamation is given, it is better to disregard Géry’s [32] observation. At least two other papers have suggested the presence of *Planaltina* in the rio São Francisco basin. Alves & Pompeu ([33], p. 592), studying the rio das Velhas fish fauna, reported a putative new species to the genus; no formal description or diagnostic characters were given. Santos *et al*. [34], who presented a checklist of the rio Pandeiros ichthyofauna, recorded *Planaltina* sp. We were unable to access the lots listed by Alves & Pompeu [33] (MZUSP 73709 and MZUSP 73785). The specimens assigned by Santos *et al*. [34] to *Planaltina* sp. (UFRGS 10158) can be assigned to *Lepidocharax* cf. *diamantina* by the following characters: base of first dorsal-fin ray slightly anterior to base of first anal-fin ray, a few apparently adnate scales on proximal portion of caudal fin, 8 scale-rows between lateral line and base of first dorsal-fin ray. They also presented 22–24 branched anal-fin rays, 4–5 teeth in the inner premaxillary series, 4–5 maxillary teeth, 17–19 circumpeduncular scale rows. Unfortunately, all five specimens are young (about 20 mm SL), which makes it difficult to give them a precise identification. If they in fact belong to *L*. *diamantina*, then the distribution of that species is wider than that reported by Ferreira *et al*. [5] (Fig 4).

### *Planaltina kaingang*, new species

**Fig 5:**
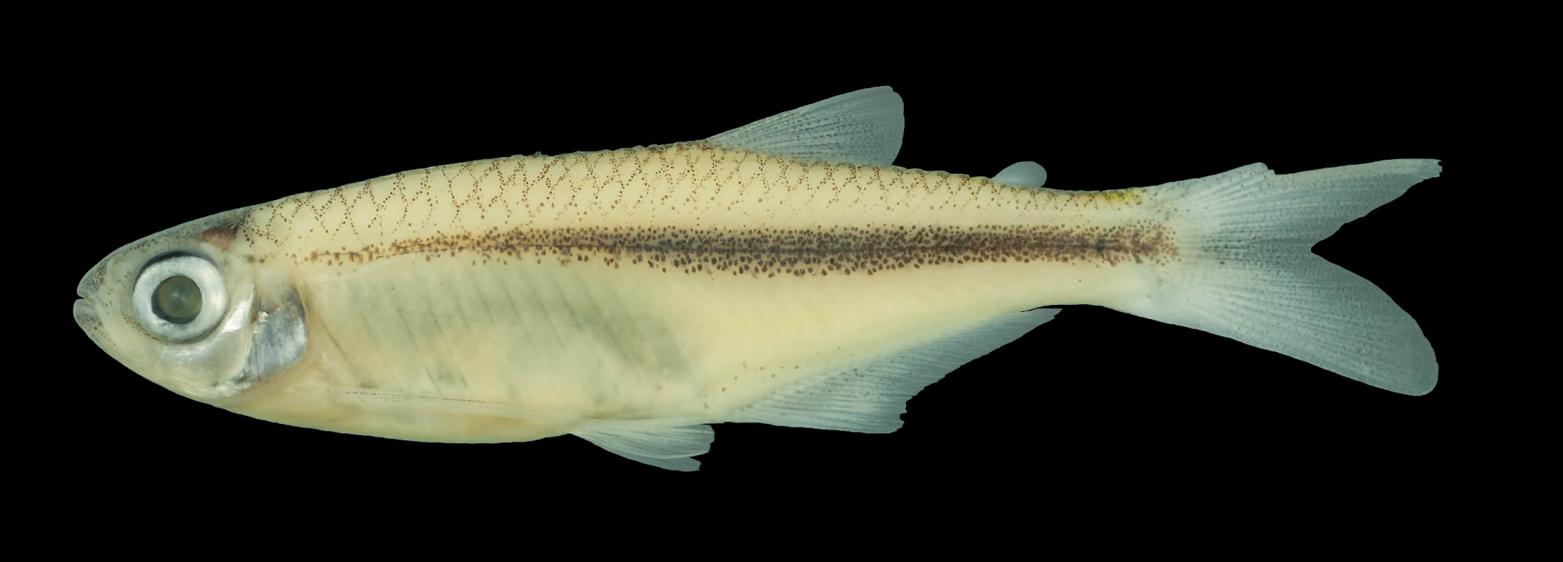
*Planaltina kaingang*, new species, holotype, MCP 50191, male, 37.9 mm SL, Paraná State, between municipalities of Marialva and Bom Sucesso, rio Keller, tributary to the rio Ivaí, upper rio Paraná.

(Fig 5, S2 Fig, Table 1)

**Table 1:** Morphometric data of *Planaltina kaingang*, new species. Based on holotype (Ht) and 24 paratypes (7 females, 17 males). X = mean; SD = standard deviation.

		Females			Males		
	Ht	Range	X	SD	Range	X	SD
Standard length	37.9	39.1–54.6	45.5	–	31.2–53.7	42.3	–
Percents of Standard Length							
Body depth at dorsal–fin origin	25.9	25.5–28.7	27.3	1.2	22.1–29.2	25.7	1.6
Snout to dorsal–fin origin	58.0	58.9–60.4	59.8	0.5	54.9–61.3	58.9	1.8
Snout to pectoral-fin origin	26.6	25.4–26.9	25.8	0.5	24.8–28.0	26.2	0.9
Snout to pelvic-fin origin	46.2	46.0–49.8	47.8	1.3	42.9–52.0	47.2	2.0
Snout to anal-fin origin	58.0	60.7–65.4	62.9	1.6	59.0–67.1	61.8	2.0
Caudal-peduncle depth	10.0	9.0–10.4	9.8	0.4	7.7–11.2	9.7	0.8
Caudal-peduncle length	15.6	11.9–14.6	13.5	0.9	10.4–16.6	13.9	1.7
Pectoral-fin length	23.2	20.9–24.3	22.5	1.3	20.2–25.6	22.3	1.3
Pelvic-fin length	13.5	11.5–14.9	13.0	1.2	12.3–14.7	13.5	0.7
Dorsal-fin base length	10.3	7.9–12.6	10.0	1.5	8.4–12.0	10.1	1.1
Dorsal-fin height	21.1	18.9–21.8	19.9	1.0	17.8–22.4	19.8	1.2
Anal-fin base length	28.5	26.5–32.5	28.4	2.0	25.2–30.3	28.3	1.7
Anal-fin lobe length	16.9	15.2–18.6	17.4	1.2	14.5–18.8	16.4	1.1
Eye to dorsal-fin origin	45.4	44.0–50.0	47.4	1.9	41.0–48.6	45.8	1.9
Dorsal-fin origin to caudal-fin base	44.3	38.5–44.2	41.6	1.8	40.4–45.1	42.7	1.5
Bony head length	22.7	22.0–23.1	22.7	0.4	21.4–24.4	22.6	0.8
Percents of Head Length							
Horizontal eye diameter	40.7	34.4–39.6	36.4	1.9	34.0–41.2	37.4	2.3
Snout length	24.4	22.0–28.0	25.6	2.5	20.6–28.0	25.1	1.9
Least interorbital width	33.7	32.8–34.9	33.4	0.8	29.2–36.0	33.0	2.0
Upper jaw length	40.7	37.4–44.8	40.8	2.5	37.1–46.9	41.7	2.4

*Planaltina* sp.–Frota *et al*. [15] (species inventory, rio Ivaí basin, upper rio Paraná).

urn:lsid:zoobank.org:act:3401E3A5-58AD-4E07-8A3B-5D3FB9AD7DF7

#### Type-specimens

Holotype. MCP 50191*, male, 37.9 mm SL, Paraná State, between municipalities of Marialva and Bom Sucesso, rio Keller, tributary to the rio Ivaí, upper rio Paraná basin, 23°38'30"S 51°51'33"W, elevation 517 m, 10 Feb 2015, G. C. Deprá, F. Souza & A. Frota.

Paratypes. All from rio Ivaí basin, upper rio Paraná basin, Paraná State, Brazil. DZSJRP 21037*, 3 females (f), 34.9–39.0 mm SL, 3 males (m), 34.6–35.0, 4 young (y) 24.2–29.6, collected with the holotype. DZSJRP 21038*, 9 f, 31.4–49.7 mm SL, 8 m, 43.2–48.2 mm SL, 7 y, 26.0–30.1 mm SL, municipality of Cândido de Abreu, rio Maria Flora, tributary to the rio Ubazinho, 24°36'32"S 51°15'31"W, elevation 573 m, 6 Apr 2014, G. C. Deprá, F. Souza & H. J. Message. MCP 50192*, 11 f, 33.0–53.6 mm SL, 6 m, 33.5–53.3 mm SL, 9 y, 28.1–31.7 mm SL, same data as DZSJRP 21038. NUP 15974, 1, 47.3 mm SL, municipality of Prudentópolis, rio Barra Grande, 24°59'35.36"S 51°9'4.38"W, elevation 532 m, 19 Jan 2014, W. J. Graça, F. A. Teixeira, R. J. Graça, W. M. Domingues. NUP 16383, 29, 16.5–47.3 mm SL, municipality of Cândido de Abreu, rio Ubazinho, 24°35'20"S 51°14'56"W, elevation 591 m, 6 Apr 2014, G. C. Deprá, F. Souza & H. J. Message. NUP 17139*, 20, 22.4–38.0 mm SL, type-locality, 25 Aug 2014, G. C. Deprá, R. R. Ota, L. F. Pesenti Júnior, N. B. Mateussi, V. N. Gomes. NUP 17152*, 70, 19.3–40.0 mm SL, collected with the holotype. UFRGS 21960*, 17 f, 33.8–53.7 mm SL, 5 m, 36.0–49.5 mm SL, 10 y, 26.7–32.8 mm SL, same data as DZSJRP 21038.

#### Non-type material

Brazil. All from the upper rio Paraná basin, Paraná State, Brazil. NUP 52, 3, 28.0–29.0 mm SL, between municipalities of Campina da Lagoa and Campo Bonito, unknown stream, tributary to the rio Piquiri, 24°43’2”S 52°55’57”W, 29 Apr 1989, Nupélia’s collecting team. NUP 16378, 26 (12 c&s, 30.7–50.5 mm SL), 23.1–50.5 mm SL, municipality of Cândido de Abreu, arroio Lajeadão, tributary to the rio Ubazinho, tributary to the rio Ivaí, 24°32'26"S 51°20'8"W, 5 Apr 2014, G. C. Deprá, F. Souza & H. J. Message. NUP 16406*, 101, 25.7–55.9 mm SL, same data as DZSJRP 21038. NUP 17323, 90, 20.7–55.5 mm SL, same locality as DZJRP 21038, 9 Mar 2013, G. C. Deprá, F. T. Mise, L. F. C. Tencatt & F. Silvério. NUP 17324, 14, 31.9–50.7 mm SL, same locality and collectors as DZSJRP 21038, 5 Apr 2014. NUP 17325, 1, 44.5 mm SL, same data as NUP 16378. NUP 17326, 3, 27.5–28.4 mm SL, collected in the type-locality, 22 Jul 2015, C. H. Zawadzki. NUP 17506, 4, 26.8–33.7 mm SL, type-locality, 7 Aug 2015, G. C. Deprá, A. Frota, F. M. Azevedo.

#### Diagnosis

**Fig 6:**
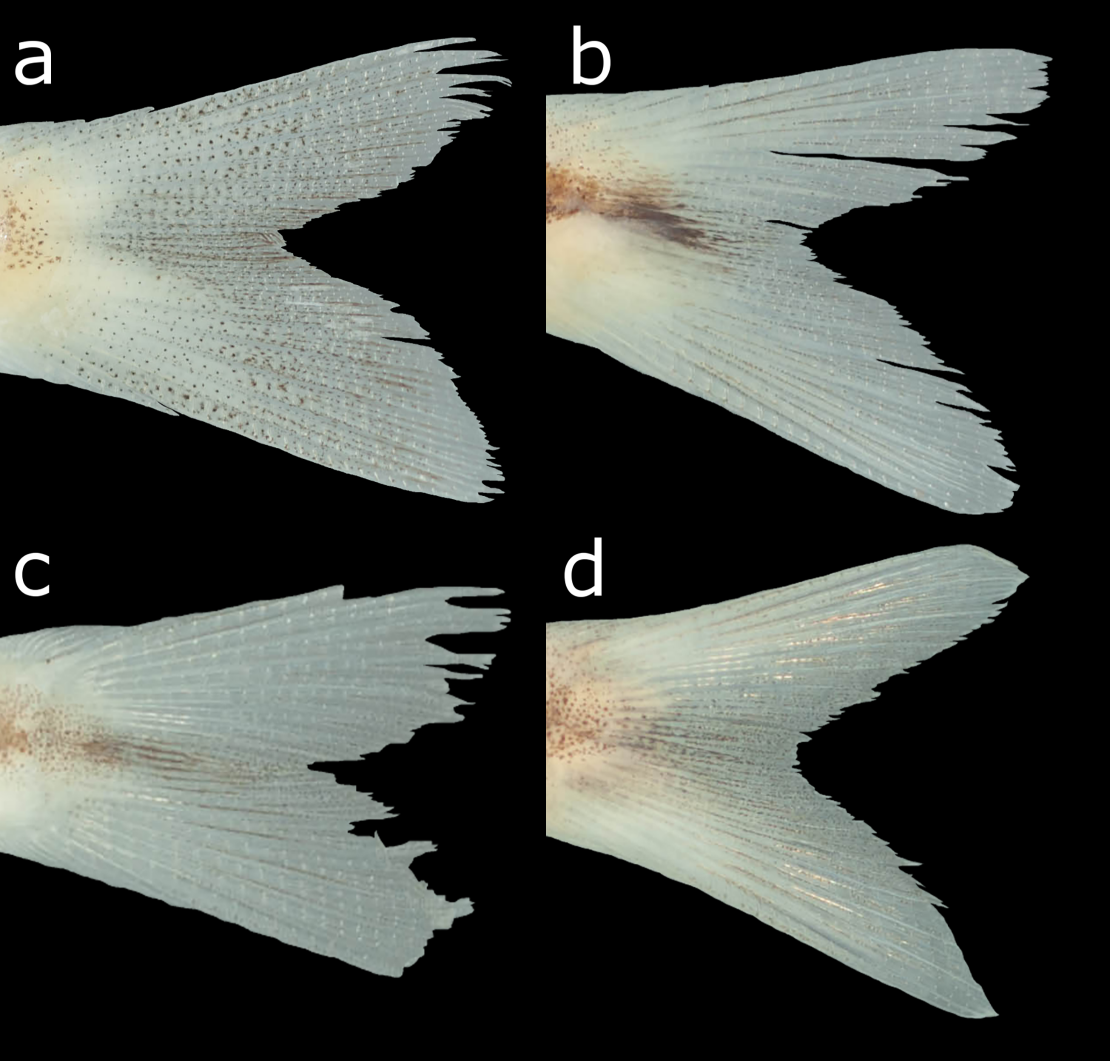
Caudal-fin melanophores of (a) *Planaltina kaingang*, new species, DZSJRP 21038, paratype, male, 47.6 mm SL; (b) *P*. *britskii*, NUP 18756, male, 33.6 mm SL; (c) *P*. *glandipedis*, LBP 14618, female, 27.7 mm SL; (d) *P*. *myersi*, NUP 18757, female, 34.5 mm SL.

**Fig 7:**
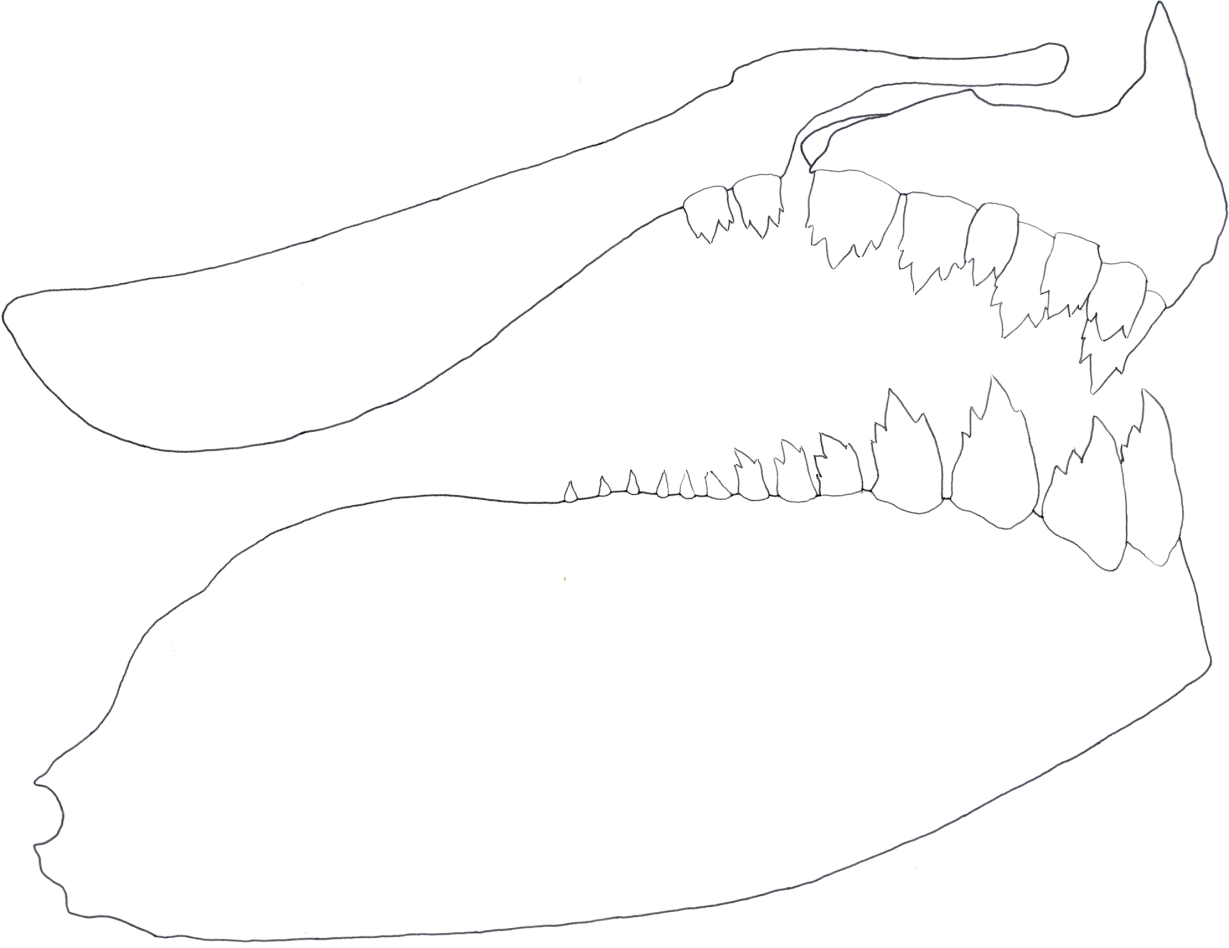
Right-side jaw dentition of *Planaltina kaingang*, new species, NUP 16378, 43.5 mm SL. Notice that bones are not shown in their natural position and the limits between the bones in the lower jaw are not depicted.

*Planaltina kaingang* is distinguished from all other congeners by the presence of melanophores embedded in all caudal-fin rays and interradial membranes (usually a few in each ray segment in specimens larger than about 25.0 mm SL) *vs*. melanophores located only on the interradial membranes, especially those between rays 8 to 15, but never embedded in the rays, regardless of SL (Fig 6). *Planaltina kaingang* is further distinguished from *P*. *britskii* by the absence of a modified, adnate scale located on the inner side of the caudal pouch of males, *vs*. presence (Fig 2A and 2B); the presence of 2–4 very elongate scales along the dorsal rim of the caudal-pouch opening in both sexes, *vs*. males always with 2 very elongate scales and females usually with only 1 (occasionally 2) enlarged, but not elongate scale (Fig 2A–2C); 15–17 circumpeduncular scale series, *vs*. 12–15. From *P*. *glandipedis*, by the presence of adipose fin, *vs*. absence; and the presence of tetra- and pentacuspid teeth in the inner series of the premaxilla and in the anterior portion of dentary (Fig 7), *vs*. all oral teeth with up to three cusps. From *P*. *myersi*, by the presence of 2–4 very elongate scales along the dorsal rim of the caudal-pouch opening in both sexes, *vs*. only one very enlarged scale in both sexes (Fig 2A and 2D); and by the presence of 2–3 distinct scales forming the anterior portion of caudal pouch opening, *vs*. anterior border of the pouch opening formed by the fusion of several scales into one big scale (Fig 2A and 2D).

#### Description

Morphometric data in Table 1. Dorsal profile slightly convex from tip of premaxilla to insertion of first dorsal-fin ray; straight from this point to adipose fin; slightly concave on caudal peduncle. Ventral profile distinctly convex from lower jaw to anal-fin origin; slightly concave on anterior half of anal-fin; slightly convex on posterior half of anal-fin; slightly concave on caudal peduncle. Insertion of first dorsal-fin ray slightly anterior to vertical through first anal-fin ray, posterior to middle of standard length; posterior end of adipose-fin base at about vertical through insertion of last anal-fin ray. Tip of longest pectoral-fin rays reaching or slightly surpassing origin of pelvic fin; tip of longest pelvic-fin rays almost reaching or slightly surpassing anal-fin origin.

Head and trunk compressed, somewhat ovoid in cross-section, broader dorsally, tapering ventrally. Pre-anal region somewhat keeled, pre-dorsal region somewhat round. Greatest width just posterior to supracleithrum. Distance between insertions of contralateral first pelvic-fin rays small, half distance between insertions of contralateral first pectoral-fin rays.

Head longer than deep. Dentary very slightly projecting anterior to premaxilla. Mouth in same horizontal as center of pupil. Tip of maxilla at vertical through anterior margin of pupil, reaching about junction of infraorbitals 2 and 3. Nostril at horizontal through dorsal margin of pupil. Eye little anterior to middle of head length, slightly dorsal to center of head depth. Infraorbital 3 touching dorsal margin of horizontal arm and anterior margin of vertical arm of preopercle. Dorsal end of gill opening in same horizontal as dorsal margin of eye.

Dorsal-fin rays ii,7,i*(21), ii,8,i(1); anal-fin rays iv,23(1), iv,24(2), v,22*(6), v,23(7), v,24(4), v,25(1), vi,23(1); pectoral-fin rays i,8,ii(2), i,8,iii*(4), i,9,i(2), i,9,ii(5), i,9,iii(3), i,10,i(4), i,10,ii(2); pelvic-fin rays, i,5,i*(21), i,6,i(1). Distal portion of medialmost pelvic-fin ray curved laterad.

Scales cycloid. Radii 3–8 in dorsal region of flank and caudal-peduncle; 4–6 in ventral region of body anterior to pectoral-fin base; 8–12 in pre-pelvic region; 1–6 in region over ribs; 4–7 between lateral line and anal-fin base; 7–11 on ventral surface of caudal peduncle. Largest modified caudal-fin scale with about 5–30 radii.

Lateral line complete, perforated scales 37(1), 38(8), 39(9), 40(13), 41(20), 42*(6), 43(1). In several specimens, last lateral-line scale represented by a single tube bearing *lateralis* canal. Scale series between lateral line and base of first dorsal-fin ray 5½*(7), 6(58), 6½(9); between lateral line and base of first anal-fin ray 3½(18), 4(40), 4½*(14), 5(1). Pre-dorsal scales 15*(13), 15½(8), 16(23), 16½(5), 17(12), 17½(2), 18(7), 18½(2), 19(1). Circumpeduncular scale-series 15(14), 16*(38), 17(7).

External gill rakers on first gill arch: 4+10(1), 5+10(2), 5+11(1), 5+12(1), 5+13(1), 6+9(1), 6+10(1), 6+11(4), 6+12(2), 6+13(1), 7+9(1), 7+11(2), 8+11(1).

Pre-maxilla with two series of teeth: outer series with 2(2), 3(4), 4*(15), 5(1) tricuspid teeth, frequently with different counts between two sides, but usually with four teeth at least in one side; inner series with 4*(22) tri- to pentacuspid teeth, but three specimens with 5 on right side. Maxilla with 1(2), 2*(18), 3(1) uni- to pentacuspid, usually tricuspid teeth. Dentary teeth usually abruptly decreasing in size posteriorly to fourth tooth: three anteriormost teeth about equal in size; fourth tooth little more than half as large as third tooth; fifth tooth usually much smaller than fourth, less than half its size; from fifth tooth to posteriormost tooth, teeth decreasing gradually in size. Two specimens presented fifth tooth little smaller than fourth, seemingly forming gradual decrease from anteriormost to posteriormost teeth. Teeth posterior to fourth tooth 6(1), 7(3), 8(1), 9(1), 10(5), 11(1), uni- to tricuspid.

Vertebrae 15(1), 16(11) abdominal; 21(3), 22(9) caudal; 37(4), 38(8) total. Ribs 11(1), 12(10), attached to 5^th^(11) to 15^th^(1), 16^th^(10) vertebrae. Supraneurals 8(4), 9(7), 10(1), comprised between 4^th^(11), 5^th^(1) and 12^th^(3), 13^th^(8), 14^th^(1) vertebrae. Dorsal-fin pterygiophores 9(13), comprised between 14^th^(7), 15^th^(5) abdominal vertebra and 4^th^(3), 5^th^(9) caudal vertebra. Anal-fin pterygiophores 23(1), 24(4), 25(6), 26(1), comprised between 1^st^(2), 2^nd^(10) and 12^th^(2), 13^th^(4), 14^th^(6) caudal vertebrae. Procurrent anal-fin rays 5(2), 6(7), 7(3), of which 0(1), 1(6), 2(4), 3(1) anterior to anal-fin pterygiophores and not attached to them. Dorsal procurrent caudal-fin rays 11(6), 12(6); ventral procurrent caudal-fin rays 10(5), 11(7).

#### Color in alcohol

Background light yellow. Infraorbital and opercular series and isthmus silvery. Otic region of skull brownish. Top of head and snout and anterior portion of lower jaw darkened by numerous, highly concentrated melanophores, especially in specimens smaller than 35 mm SL. Melanophores on midline of dorsum forming thin stripe from tip of supraoccipital to caudal-fin base. On remainder of dorsal region of flank and caudal peduncle, melanophores concentrated on scale margins, forming reticulate pattern. Dark longitudinal stripe from just dorsal to gill opening to base of caudal-fin rays; centered on horizontal myoseptum, along which melanophores concentration is highest; about one-scale deep from about vertical through base of first dorsal-fin ray to vertical through adipose fin, broadening slightly towards end of caudal peduncle, tapering anteriorly to vertical through first dorsal-fin ray. Longitudinal stripe occasionally covered by silvery pigmentation, partially hiding melanophores. In humeral region, melanophores of longitudinal stripe scattered, never forming spot. Melanophores absent or scarcely concentrated immediately dorsal to longitudinal stripe, leaving clear area between reticulated area dorsally and longitudinal stripe. Ventral to it, melanophores almost absent, except for a few scattered over anal-fin pterygiophores. All fins with hyaline background. Dorsal fin with melanophores on interradial membranes only, mostly edging rays. Adipose fin usually with no melanophores, rarely with a few on its anterior margin. Caudal fin with melanophores scattered over interradial membranes and embedded in rays; interradial membranes of middle rays usually with higher concentration of melanophores, but not continuing longitudinal stripe of flank; melanophores embedded in caudal-fin rays usually one in each segment, occasionally none or more than one. Anal fin with melanophores only on interradial membranes, as in dorsal fin. Pelvic fin usually with no melanophore, rarely a few embedded only in first ray. Pectoral fin with no melanophores or with some embedded in first one to three rays.

#### Color in life

See S2 Fig. Head silvery. Trunk somewhat translucent, with silvery glow; peritoneum silvery; lateral band distinct, but completely covered by silver pigment, hiding black pigment underneath. Melanophores evident on base of median caudal-fin rays and distal portion of all caudal-fin rays.

#### Sexual dimorphism

Males with hooks on anal and pelvic fins and fusion of anteriormost external ceratobranchial 1 gill filaments; pelvic-fin length apparently unrelated to sex (Table 1). Anal-fin hooks from longest unbranched to 9^th^ or 10^th^ branched ray, always 1 on each ray segment, raising from posterior margin of ray or ray branches, laterally directed (but tip directed to base of ray when hook well developed). One to 7 hooks distributed along middle to distal portion of longest unbranched ray. Usually 2–4 hooks on branched rays. First branched-ray hooks usually on proximal segments of posterior branch, but in occasional specimens also present in one or two segments immediately proximal to ramification. Second to fifth branched-ray hooks usually on proximal segments of posterior branch, but some specimens with fewer poorly developed hooks on anterior branch. Sixth to ninth or tenth branched-ray hooks on segments immediately proximal to ramification. Pelvic-fin hooks lacking on first (unbranched) ray, but occasionally present in each of remaining rays; always one on each segment, raising from medial margin of ray or ray branches, medially directed (but tip directed to base of ray when hook well developed). Each ray with up to eleven hooks from proximal region to almost tip of medial branch and only a few poorly developed ones on lateral branch, if any. Gill filaments incompletely fused, attached to one another but still seen as separate units externally.

#### Distribution

*Planaltina kaingang* is known from the rio Keller, from the rio Ubazinho basin and from the rio Barra Grande, all immediate tributaries to the rio Ivaí; and from the rio Piquiri, upper rio Paraná basin, Paraná State, Brazil (Fig 4).

#### Ecology and habitat

*Planaltina kaingang* was collected in streams about 8–10 m wide, a few centimeters to little more than 1 m deep. A few specimens were collected in streams with bedrock bottom (*e*. *g*., arroio Lajeadão and rio Keller a few kilometers downstream from the stretch where the holotype was captured), more specimens were collected over fine gravel (rio Keller where the holotype was captured), but their abundance was by far the greatest in the rio Maria Flora, on sandy bottom, about 1.2 m deep. This locality also yielded the largest specimens. *Planaltina kaingang* was captured mostly close to the water surface, where it probably forages on allochthonous insects, which were found in some stomachs observed. *Planaltina kaingang* in the rio Keller was collected along with *Piabarchus* aff. *stramineus* Eigenmann (a species to which it is overwhelmingly similar, also lacking a humeral mark), apparently forming a single school in which the latter was more abundant. In the rio Maria Flora, *P*. *kaingang* was by far the most abundant species and was caught along with *Oligosarcus* cf. *paranensis* Menezes & Géry (small-sized individuals) and *Bryconamericus coeruleus* Jerep & Shibatta, which has a subterminal mouth and is also abundant. The maximum size reached by *P*. *kaingang* is evidently superior to that observed for other congeners, reaching up to 54.6 (female) and 53.7 (male) mm SL. The smallest males of *P*. *kaingang* observed to present fully developed hooks are about 32.1 mm SL. The maximum SL for each species was as follows: *P*. *britskii*, 37.4 (female) and 37.4 mm SL (male; fully developed hooks already at 26.0 mm SL); *P*. *glandipedis*, 30.4 (female) and 29.5 mm SL (male; fully developed hooks already at 25.6 mm SL); *P*. *myersi* from the rio Paranaíba basin, 36.6 (female) and 36.7 mm SL (male; fully developed hooks already at 29.8 mm SL); and *P*. *myersi* from the rio São Francisco basin, 42.1 (female) and 31.1 mm SL (male; fully developed hooks already at 28.4 mm SL).

#### Etymology

*Planaltina kaingang* is named after the Kaingang, an ethnic group that have been inhabiting the southern portion of Brazil for centuries, including some areas in the vicinities of the rio Ubazinho drainage, where the new species was first recognized.

### Key to the species of *Planaltina*

1. Adipose fin absent; all premaxillary and dentary teeth with up to three cusps P. glandipedis

1’. Adipose fin present; at least some of the inner-series premaxillary teeth and anterior dentary teeth tetra- and pentacuspid 2

2. A single, very elongate scale forming the dorsal margin of the caudal pouch opening in both males and females; a complex of fused scales forming the anterior margin of the caudal pouch opening (Fig 2D) *P*. *myersi*

2’. More than one scale forming the dorsal margin of the caudal pouch opening (except female of *P*. *britskii*, but then scale is not elongate); no fused scales in the anteroventral margin of the pouch (Fig 2A–2C) 3

3. Melanophores present on some caudal-fin interradial membranes, but absent from caudal-fin rays (Fig 6B); males with two scales forming the dorsal margin of the caudal pouch opening and one adnate scale forming the medial wall of the pouch (Fig 2B), females with only one scale (rarely two) forming the dorsal margin of the caudal pouch opening, not elongate (Fig 2C); 12–15 (rarely 15) circumpeduncular scale rows P. britskii

3’ Melanophores embedded in all caudal-fin rays and interradial membranes (Fig 6A); 2–4 elongate scales forming the dorsal margin of the caudal pouch opening regardless of gender (Fig 2A); 15–17 circumpeduncular scale rows *P*. *kaingang*

### Comments on *Lepidocharax burnsi*

**Fig 8:**
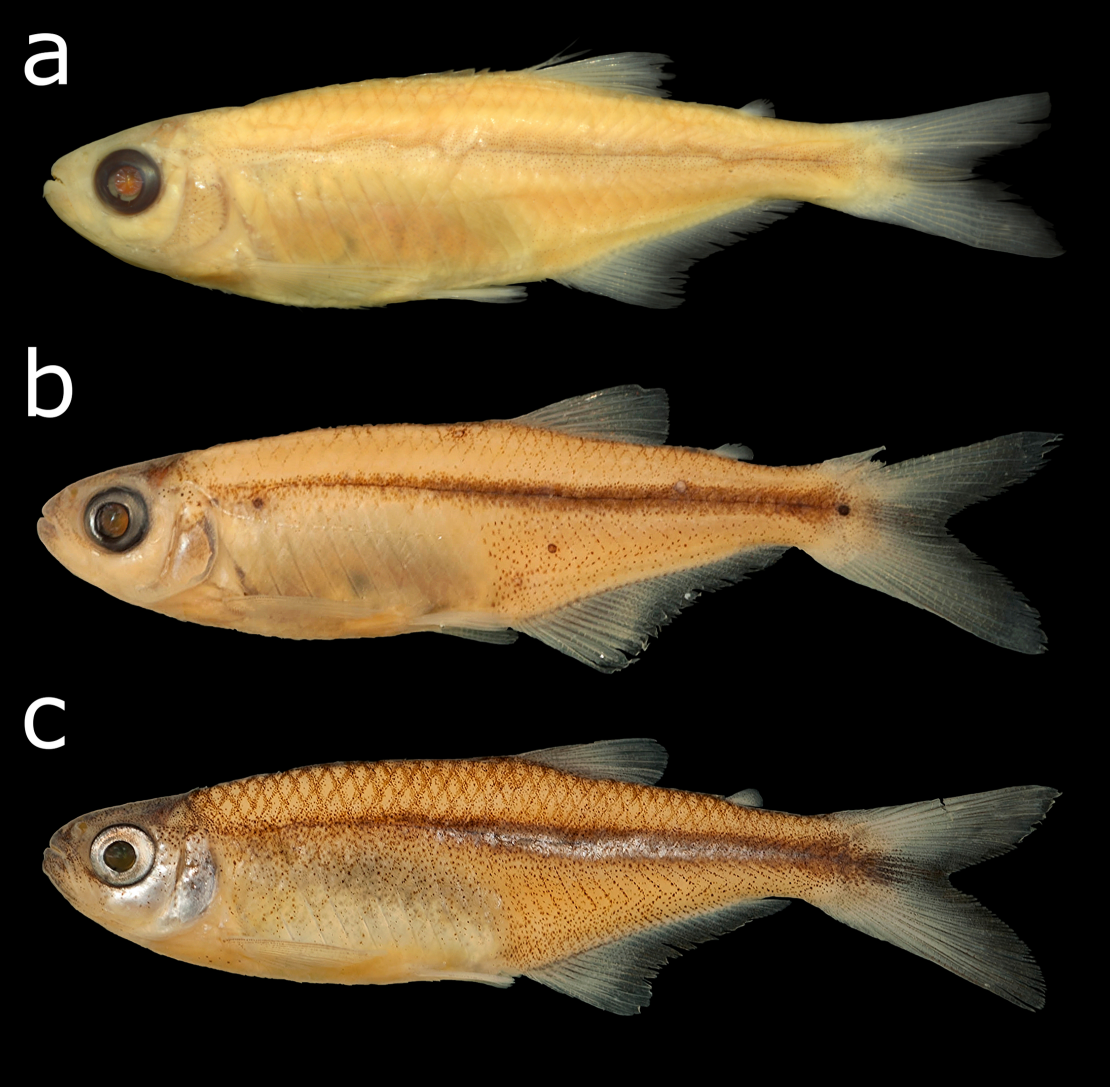
*Lepidocharax burnsi*: (a) holotype, MCP 45718, female, 34.7 mm SL; (b) paratype, MCP 27829, 27.8 mm SL; (c) non-type, UFRGS 11275, 33.4 mm SL.

The specimen of *Lepidocharax burnsi* depicted by Ferreira *et al*. [5] is not the holotype, as the caption claims, but the paratype MCP 31798, which was collected along with the holotype (MCP 45718). All of the morphometric and meristic data presented by Ferreira *et al*. [5] as belonging to the holotype has, however, been checked and considered to be correct. A picture of the true holotype is given in Fig 8A. Both specimens are very badly preserved regarding their color patterns, but the MCP 31798 specimen also seems to present a malformation in the snout, which allowed us to identify the mistake. The holotype and especially the remainder of the paratypes and the non-type specimens look exactly like a *Planaltina* species regarding color pattern and body shape (Fig 8). However, as mentioned before, they differ by the caudal-fin squamation pattern.

## Discussion

### Geographic distribution

Despite all species of the genus inhabit the upper rio Paraná basin, the geographic distribution of *Planaltina* species is basically allopatric: *P*. *kaingang* was found in the Ivaí and Piquiri river basins, and *P*. *myersi* in the rio Paranaíba basin; both the Ivaí and Paranaíba drainages have been considered as endemic regions by previous authors [15, 35]. The rivers draining the cuestas of the State of São Paulo, where *P*. *glandipedis* is endemic, have also been suggested to have their own exclusive species [36].

Regarding the endemism in the rio Ivaí basin, Frota *et al*. [15] considered 12 fish species to be exclusive to it, out of 100 native species. Evidence gathered posteriorly shows that, in addition to *Planaltina* sp. (= *P*. *kaingang*), at least *Bryconamericus* sp. (= *B*. *coeruleus*) is not endemic, thus the rio Ivaí basin totals 10 endemic species (10%). In comparison, the rio Tibagi has about 110 native species [37], of which only three are endemic (less than 3%), all loricariids: *Isbrueckerichthys calvus* Jerep, Shibatta, Pereira & Oyakawa [38], *Isbrueckerichthys saxicola* Jerep, Shibatta, Pereira & Oyakawa [38] and *Neoplecostomus yapo* Zawadzki, Pavanelli & Langeani [39].

The rate of endemism in the rio Ivaí is equivalent to that of the rio Jordão, in which 11% of native species are endemic [40]. In spite of being much smaller, the rio Jordão is part of the lower rio Iguaçu basin, where major adaptive radiations took place due to a series of waterfalls that isolated its tributaries from one another and from the rio Paraná basin [41, 42]. Species from the rio Ivaí, on the other hand, are not geographically restricted by self-evident barriers. This is curious, because there is noticeably a number of species that are present in the upper rio Paraná floodplain and widespread in the upper rio Paraná basin, but not in the rio Ivaí basin (except, in some cases, in the very lower portion). Some examples are *Aphyocheirodon hemigrammus*, *Cyphocharax vanderi*, *Steindachnerina insculpta*, *Leporellus* cf. *vittatus*, *Schizodon altoparanae*, *Microglanis garavelloi*, *Rhyacoglanis paranensis* and *Piabarchus stramineus* (*P*. aff. *stramineus*, a species without humeral spot and a diffuse spot on caudal fin, is present). This suggests a double-way barrier, whose nature is unknown, preventing some species to disperse upstream, and others to disperse downstream.

### Diagnostic characters

*Planaltina* species, other than *P*. *kaingang*, have the caudal fin roughly hyaline except for the median portion, through which the lateral band of the flank continues posteriorly almost to the tip of the rays; a close observation reveals that melanophores may be present throughout the fin, usually bordering the fin rays, never on the rays themselves. In *P*. *kaingang*, melanophores are more homogeneously distributed, even embedded in the fin rays, and, to the naked eye, the caudal fin looks dusky. This character was analyzed in a very large sample and is conservative among *Planaltina* species, thus permitting the ready recognition of the new species. Among other Stevardiinae available to the study, those presenting the most similar condition to *P*. *kaingang* are *Diapoma speculiferum*, *D*. *terofali* and *Mimagoniates microlepis* (Steindachner). In those species, there are usually one or two melanophores in each ray segment throughout the caudal fin, but they are also spread on the interradial membranes and not particularly concentrated along the borders of the rays. The latter condition, in which the melanophores are united along the borders of the rays, forming thin black lines, is evident in, *e*.*g*., *Bryconamericus exodon* Eigenmann (especially on distal portion of caudal-fin lobes), *B*. aff. *iheringii* (Boulenger), *B*. *ikaa* Casciotta, Almirón & Azpelicueta [43], *B*. *patriciae* Silva, *B*. *pyahu* Azpelicueta, Casciotta & Almirón [44], *B*. *turiuba* Langeani, Lucena, Pedrini & Pereira [45], *Caiapobrycon tucurui* Malabarba & Vari ([46], Figs 2 and 3), *Creagrutus meridionalis* Vari & Harold [47], *Diapoma* aff. *alburnus* (Hensel), *Hemibrycon surinamensis* Géry, *Knodus moenkhausii*, *Knodus victoriae* (Steindachner), *Piabarchus stramineus*, *P*. aff. *stramineus* (*sensu* Frota *et al*. [15]) and *Piabina argentea* Reinhardt. Occasional melanophores embedded in caudal-fin rays appear in specimens of most aforementioned species, but rarely in all segments of all fin rays, and they are usually more concentrated along the margins of the rays. In any case, this character seems to be less conservative in those species than in *Planaltina* species. *Lepidocharax burnsi* presented a pattern similar to that of *P*. *britskii*, *P*. *glandipedis* and *P*. *myersi*.

The caudal-fin squamation pattern, which is important to diagnose *Planaltina* species, is very conservative as well. Both *P*. *britskii* and *P*. *myersi* exhibit conditions that are probably autapomorphic: *P*. *britskii* is the only species in Diapomini to present a sexually dimorphic caudal-fin squamation, and *P*. *myersi* is the only one to exhibit a fusion of scales surrounding the opening of the caudal pouch. Only *P*. *glandipedis* presents a similar squamation in comparison to *P*. *kaingang*, but the two species are easily distinguished by the presence or absence of the adipose fin and by the dentition. Both characters are invariable in the specimens examined herein.

### Sexual dimorphism

Sexual dimorphism in *Planaltina* species is homogeneous regarding fin hooks (see Menezes *et al*. [10]). Gill glands, in contrast, were observed herein in the new species and also in all males of *P*. *britskii* and *P*. *myersi*, but not in *P*. *glandipedis*, despite the fact that several hook-bearing individuals were analyzed for it. However, it is also possible that the gland is present in *P*. *glandipedis*, but much less developed than in the other species (see Terán *et al*. [48] for the presence of underdeveloped glands in some species of *Astyanax* Baird & Girard). Burns and Weitzman [49] first described a similar organ in *Corynopoma riisei* Gill, and posterior papers described it for several other characids [48, 50]. Mirande [2] was the first to employ this character in a phylogenetic analysis of the Characidae, finding it to have been independently acquired at least two times and lost a few times throughout the evolution of the group. Herein this character is better understood as reversed in *P*. *glandipedis*. Sexual dimorphism is otherwise found in *Planaltina* only in the caudal-fin squamation of *P*. *britskii*, as commented above.

## Comparative material

All from Brazil. *Bryconamericus exodon*. NUP 13341, 26, 22.5–47.0 mm SL, rio Paraguai basin, 20°19'37"S 55°13'23"W. *Bryconamericus* aff. *iheringii*. NUP 7998, 6, 69.0–80.2 mm SL, rio Tibagi basin, 25°1'34"S 50°3'58"W. *Bryconamericus ikaa*. NUP 4341, 20, 46.8–58.0 mm SL, rio Iguaçu basin, 25°30'49"S 53°0'4"W. *Bryconamericus patriciae*. NUP 16278, 26, 28.2–63.9 mm SL, rio Uruguai basin, 28°26'38"S 50°6'30"W. *Bryconamericus pyahu*. NUP 7310, 7, 25.0–46.0 mm SL, rio Iguaçu basin, 25°32'12"S 53°29'11"W. *Bryconamericus turiuba*. NUP 6170, 9, 46.6–55.3 mm SL, rio Iguatemi (upper rio Paraná basin), 23°14'60"S 55°31'0"W. *Caiapobrycon tucurui*. NUP 8999, 8, 21.0–28.0 mm SL, rio Araguaia basin, 6°17'25"S 48°27'9"W. *Creagrutus meridionalis*. NUP 8448, 4, 20.5–61.5 mm SL, rio Paraguai basin, 17°37'42"S 52°28'8"W. *Diapoma* aff. *alburnus*. NUP 11174, 44, 48.6–57.0 mm SL, rio Iguaçu basin, 25°32'3"S 52°59'9"W. *Diapoma speculiferum*. UFRGS 14273, 4, 43.8–45.1 mm SL, lagoa dos Patos basin, 29°21'44"S 52°7'37"W. *Diapoma terofali*. UFRGS 8368, 4, 38.8–46.9 mm SL, rio Uruguai basin, 31°36'51"S 54° 8'40"W. *Hemibrycon surinamensis*. NUP 8396, 1, 78.0 mm SL, rio Araguaia basin, 6°31'55"S 48°36'34"W. *Knodus moenkhausii*. NUP 6522, 28, 23.1–39.2 mm SL, upper rio Paraná basin, 22°45'3"S 53°15'58" W. *Knodus victoriae*. NUP 16309, 42, 25.0–54.0 mm SL, rio Parnaíba basin, 9°0'53"S 45°55'47"W. *Lepidocharax burnsi*. All from rio São Francisco basin, Minas Gerais State. MCP 27821, 20, 24.0–30.4 mm SL, municipality of Vazante, córrego Jaburu, tributary to the córrego Indaiazinho, tributary to the Ribeirão Santa Catarina, tributary to the rio Paracatu, 18°3'26"S 46°52'15"W, elevation 681 m, 21 Jan 2001, C. Lucena, J. Silva, E. Pereira & A. Cardoso. MCP 27824, 1, 30.2 mm SL, paratype, municipality of Paracatu, córrego Rico, tributary to the rio Paracatu, 17°18'16"S 46°46'14"W, elevation 592 m, 24 Jan 2001, C. Lucena, J. Silva, E. Pereira & A. Cardoso. MCP 27829, 126 (20, 24.7–29.7 mm SL), paratypes, municipality of Vazante, córrego Pirapetinga, tributary to the rio Claro, tributary to the ribeirão Arrenegado, tributary to the rio Escuro, tributary to the rio Paracatu, 17°56'39"S 46°58'9"W, elevation 625 m, 25 Jan 2001, C. Lucena, J. Silva, E. Pereira & A. Cardoso. MCP 27830, 3, 25.2–28.2 mm SL, same data as MCP 27824. MCP 31798*, 1, 36.0 mm SL), paratype, municipality of Brumadinho, rio Paraopeba, 20°9'S 44°10'W, Apr 1997, V. Vono & C. B. M. Alves. MCP 45718*, 34.7 mm SL, holotype, same data as MCP 31798. NUP 7273, 16, 27.5–29.5 mm SL, between municipalitys of Augusto de Lima and Santo Hipólito, rio Pardo, tributary to the rio das Velhas, 18°13'40"S 44°13'30"W, elevation 524 m, 18 Sep 2007, C. G. Leal. UFRGS 9870, 4, 20.4–28.4 mm SL, municipality of Bocaiúva, rio Guavinipã, tributary to the rio Jequitaí, 17°6'35"S 43°57'21"W, elevation 637 m, 23 May 2008, T. Carvalho & F. Jerep. UFRGS 9996, 4, 26.2–32.9 mm SL, municipality of Francisco Dumont, riacho da Água Fria, tributary to the rio Jequitaí, 17°17'56"S 44°21'40"W, elevation 590 m, 22 May 2008, T. Carvalho & F. Jerep. UFRGS 9999, 20, 24.4–30.6 mm SL, municipality of Várzea da Palma, ribeirão do Corrente, tributary to the rio das Velhas, 17°27'37"S 44°41'1"W, elevation 507 m, 22 May 2008, T. Carvalho & F. Jerep. UFRGS 11263, 3, 31.8–35.0 mm SL, municipality of Unaí, córrego Buritizinho, tributary to the córrego Pindaíba, tributary to the ribeirão Canabrava, tributary to the rio Preto, tributary to the rio Paracatu, 16°13'31"S 46°40'25"W, elevation 688 m, 29 Sep 2009, F. Carvalho & V. Bertaco. UFRGS 11275, 20, 23.2–35.9 mm SL, municipality of Unaí, córrego Extrema, tributary to the rio Canabrava, tributary to rhe rio Preto, tributary to the rio Paracatu, 16°9'24"S 46°44'48"W, elevation 651 m, 29 Sep 2009, F. Carvalho & V. Bertaco. *Lepidocharax* cf. *diamantina*. UFRGS 10158, 14.9–19.3 mm SL, Brazil, Minas Gerais, municipality of Bonito de Minas, unknown tributary of the rio Catolé, tributary to the rio Pandeiros, tributary to the rio São Francisco, approx. 15°17'S 44°49'W, elevation 611 (approx.), 4 Jul 2008, J. A. Dergam & U. Santos. *Markiana nigripinnis*. NUP 18758, 1, 73.6 mm SL, rio Paraguai basin, 21°46'0"S 57°55'8"W. *Mimagoniates microlepis*. NUP 9576, 10, 27.6–42.8 mm SL, rio Xaxim basin (draining into the baía de Antonina, Atlantic Ocean), 25°21'59"S 48°49'59"W. *Piabarchus stramineus*. NUP 4599. *Piabarchus* aff. *stramineus*. NUP 6536; NUP 17667. *Piabina argentea*. NUP 6928. *Planaltina britskii*. All from upper rio Paraná basin. Mato Grosso do Sul State. NUP 17320, 1 female, 32.5, between municipalitys of Naviraí and Taquarussu, rio Ivinhema, tributary to the rio Paraná, 22°59'10"S 53°39'2"W, elevation 235 m, Aug 2013, Nupélia’s collecting team. NUP 17321, 1 female, 30.8 mm SL, municipality de Ribas do Rio Pardo, rio Verde, tributary to the rio Paraná, 20°23'27"S 52°57'2"W, 439 m, Augusto Frota. NUP 17322, 1 young, 21.1 mm SL, same data as NUP 17320, 19 Nov 2014, collecting team of Nupélia’s ichthyoplancton lab. Paraná State. NUP 11802, 1 female, 27.0, 3 males, 26.4–28.1 mm SL, municipality of Porto Rico, lagoa do Genipapo, Porto Rico fluvial island, rio Paraná, 22°45'33"S 53°16'5"W, elevation 238 m, 15 Apr 2014, Nupélia’s collecting team. NUP 17702, 2, 24.0–24.5 mm SL, municipality of São Pedro do Paraná, ribeirão São Pedro, tributary to the rio Paraná, 22°44'59"S 53°13'24"W, elevation 243 m, 13 Nov 2014, C. S. Pavanelli. São Paulo State. DZSJRP 5385, 1 male, 31.9 mm SL, 2 young, 23.4–24.6 mm SL, between municipalitys of Nova Aliança and Potirendaba, ribeirão do Borá, tributary to the rio do Cubatão (= rio da Barra Mansa), tributary to the Usina Hidrelétrica Mario Lopes Leão (= Usina de Promissão) reservoir, rio Tietê, 21°1'20"S 49°27'34"W, elevation 424 m, 24 Feb 2003, J. P. Serra. DZSJRP 10242, 3 females, 29.9–31.6 mm SL, same locality as DZSJRP 5385, 29 Sep 2006, L. Casatti. DZSJRP 10617, 1 female, 31.5 mm SL, between municipalitys of Guzolândia and Santo Antônio do Aracanguá, córrego das Cobras, tributary to the ribeirão do Barreiro, tributary to the Usina Hidrelétrica de Três Irmãos reservoir, rio Tietê, 20°43'5"S 50°44'51"W, elevation 384 m, 2 May 2007, F. B. Tereza & J. L. Veronezzi. DZSJRP 10964*, 1 female, 32.9 mm SL, 2 males, 31.9–33.3 mm SL, municipality of Glicério, córrego Caximba, tributary to the ribeirão Bonito, tributary to the Usina Hidrelétrica de Nova Avanhandava reservoir, rio Tietê, 21°17'21"S 50°10'29"W, elevation 389 m, 31 Jan 2008, F. B. Tereza. DZSJRP 12734, 1 male, 26.0 mm SL, municipality of Rio Claro, ribeirão Claro, tributary to the rio Corumbataí, tributary to the rio Piracicaba, tributary to the Usina Hidrelétrica de Barra Bonita reservoir, rio Tietê, 22°21'36"S 47°30'46"W, elevation 598 m, 1 Jul 2002, A. T. B. Santos. DZSJRP 13612, 1 male, 29.6 mm SL, municipality of Salto Grande, lagoa Cava, ribeirão dos Bugres, tributary to the Salto Grande reservoir, rio Paranapanema, 22°53'21"S 49°58'30"W, elevation 390 m, unknown date and collector. DZSJRP 18192, 3 females, 29.6–31.2 mm SL, 1 male, 32.7 mm SL, municipality of Ipiguá, córrego da Barra Funda, tributary to the ribeirão da Barra Grande, tributary to the rio Preto, tributary to the rio Turvo, tributary to the rio Grande, 20°36'33"S 49°25'13"W, elevation 455 m, 8 Oct 1987, V. Garutti. MZUEL 11777, 1 female, 37.4 mm SL, 3 males, 30.2–32.4 mm SL, municipality of Salto Grande, rio Pardo, tributary to the reservoir of the UHE Salto Grande, rio Paranapanema, 22°54'16"S 49°57'6"W, elevation 385 m, 5 Nov 2013, M. Orsi. MZUSP 62760*, paratypes, 1 female, 32.7 mm SL, 1 male, 32.5 mm SL, municipality of Auriflama, córrego Limoeiro, tributary to the rio São José dos Dourados, tributary to the Usina Hidrelétrica de Ilha Solteira reservoir, rio Paraná, municipality de Auriflama-SP. NUP 18756 (ex-DZSJRP 15182), 1 female, 34.6 mm SL, 13 males, 30.8–37.4 mm SL, municipality of Bady Bassitt, córrego do Boi, tributary to the ribeirão ribeirão do Borá, tributary to the rio do Cubatão (= rio da Barra Mansa), tributary to the Usina Hidrelétrica Mario Lopes Leão (= Usina de Promissão) reservoir, rio Tietê, 20°58'14"S 49°25'23"W, elevation 440 m, 5 Oct 2011, A. R. Manzotti. *Planaltina glandipedis*. All from São Paulo State, upper rio Paraná basin. LBP 14618, 14 females, 26.1–30.4 mm SL, 8 males, 27.6–29.5 mm SL, between the municipalitys of Botucatu and São Manuel, rio Araquá, tributary to the Usina Hidrelétrica de Barra Bonita reservoir, rio Tietê, elevation 476 m, unknown date and collector. LBP 19792, 1 female, 29.8 mm SL, municipality de Avaré, rio Novo, tributary to the rio Pardo, tributary to the Usina Hidrelétrica de Salto Grande reservoir, rio Paranapanema, 23°1'27"S 48°49'41"W, elevation 701 m, unknown date and collector. MZUSP 63690*, paratype, 1 male, 25.6 mm SL, municipality of Brotas, unknown locality, tributary to the rio Jacaré-Pepira, tributary to the Usina Hidrelétrica de Ibitinga reservoir, rio Tietê, unknown coordinates and date, W. Barrela. *Planaltina myersi*. Distrito Federal. Upper rio Paraná basin. DZSJRP 10802, 1 male, 36.7 mm SL, same locality as NUP 18755, 25 Apr 2007, CIUNB. DZSJRP 10832, 1 young, 28.2 mm SL, Parque Nacional de Brasília, municipality of Brasília, ribeirão do Torto, tributary to the Paranoá reservoir, rio Paranoá, tributary to the rio São Bartolomeu, tributary to the rio Corumbá, tributary to the rio Paranaíba, 15°41'57"S 47°54'23"W, elevation 1112 m, 26 Apr 2007, CIUNB. MZUSP 36614*, 4, 34.1–35.7 mm SL, municipality of Brasília, córrego Taboca, tributary to the rio São Bartolomeu, tributary to the rio Corumbá, tributary to the rio Paranaíba, unknown coordinates and date, M. Ribeiro & J. Dalmácio. NUP 18755 (ex-DZSJRP 9975), 4 females, 32.8–36.6 mm SL, 9 males, 29.8–36.0 mm SL, 2 young, 24.7–26.6 mm SL, Parque Nacional de Brasília, municipality of Brasília, ribeirão Bananal, tributary to the Paranoá reservoir, rio Paranoá, tributary to the rio São Bartolomeu, tributary to the rio Corumbá, tributary to the rio Paranaíba, 15°43'43"S 47°54'37"W, elevation 1084 m, 12 Jan 2007, P. D. Podestá & A. Max. Upper rio São Francisco basin. DZSJRP 14261*, 1 female, 32.4 mm SL, 1 male, 30.7 mm SL, 1 young 24.8 mm SL, municipality de Brasília, ribeirão Jacaré (= córrego do Meio), tributary to the rio Preto, tributary to the rio Paracatu, tributary to the rio São Francisco, 15°37'34"S 47°23'40"W, elevation 1029 m, 23 Oct 2010, P. P. Aquino. DZSJRP 14275, 1 female, 30.0 mm SL, 1 young, 28.1 mm SL, between municipalitys of Brasília and Formosa (Goiás State), ribeirão Santa Rita, tributary to the rio Preto, tributary to the rio Paracatu, tributary to the rio São Francisco, 15°34'47"S 47°21'21"W, elevation 999 m, 23 Sep 2010, P. P. Aquino. DZSJRP 14281, 3 females, 33.9–41.2 mm SL, municipality of Brasília, ribeirão Extrema, afluente do rio Preto, afluente do rio Paracatu, afluente do rio São Francisco, 15°50'46"S 47°23'7"W, elevation 1084 m, 23 Sep 2010, P. P. Aquino. Goiás State. Upper rio Paraná basin. DZSJRP 20402, 1 young, 25.1 mm SL, municipality of Anhanguera, rio Paranaíba, 18°20'47"S 48°13'30"W, elevation 729 m, 16 Aug 2014, F. R. Carvalho & A. C. Santos. Upper rio São Francisco basin. NUP 1118, 19, 24.7–32.4 mm SL, between municipalitys of Caldas Novas and Ipameri, Usina Hidrelétrica de Corumbá reservoir, rio Corumbá, tributary to the rio Paranaíba, 9 Apr 1999, Nupélia’s collecting team. NUP 18757* (ex-DZSJRP 14255), 3 females, 29.7–34.5 mm SL, 5 males, 28.4–31.1 mm SL, 2 young, 21.6–24.0 mm SL, municipality of Formosa, córrego Santo Inácio, tributary to the rio Bezerra, tributary to the rio Preto, tributary to the rio Paracatu, tributary to the rio São Francisco, 15°49'31"S 47° 8'47"W, elevation 924 m, 18 Nov 2010, P. P. Aquino. Minas Gerais State. DZSJRP 15799, 1 young, 27.9 mm SL, municipality of Indianópolis, brook in the road to the rio Araguari ferryboat, tributary to the Miranda reservoir, rio Araguari, tributary to the rio Paranaíba, 19° 3'36"S 47°56'11"W, elevation 739 m, 10 Mar 2012, F. Langeani.

## Supporting information

S1 Fig Phylogenetic analysis. Best maximum likelihood tree showing the relationships among Triportheidae, Gasteropelecidae, Bryconidae, Acestrorhynchidae, Iguanodectidae, and Characidae. Chalceidae was used as outgroup.
(PNG)

S2 Fig *Planaltina kaingang* in life. *Planaltina kaingang*, new species, non-type specimen in life, NUP 16406, municipality of Cândido de Abreu, rio Maria Flora, rio Ivaí basin, upper rio Paraná.
(TIFF)

S1 File Sequences of primers used in present study. (DOCX)
