# Supplementary figures and images for: Molecular phylogeny of Planaltina Böhlke (Characidae: Stevardiinae) and comments on the definition and geographic distribution of the genus, with description of a new species
Source: PLoS One. 2018 May 16;13(5):e0196291. doi: 10.1371/journal.pone.0196291 (PMC5955486; doi:10.1371/journal.pone.0196291)

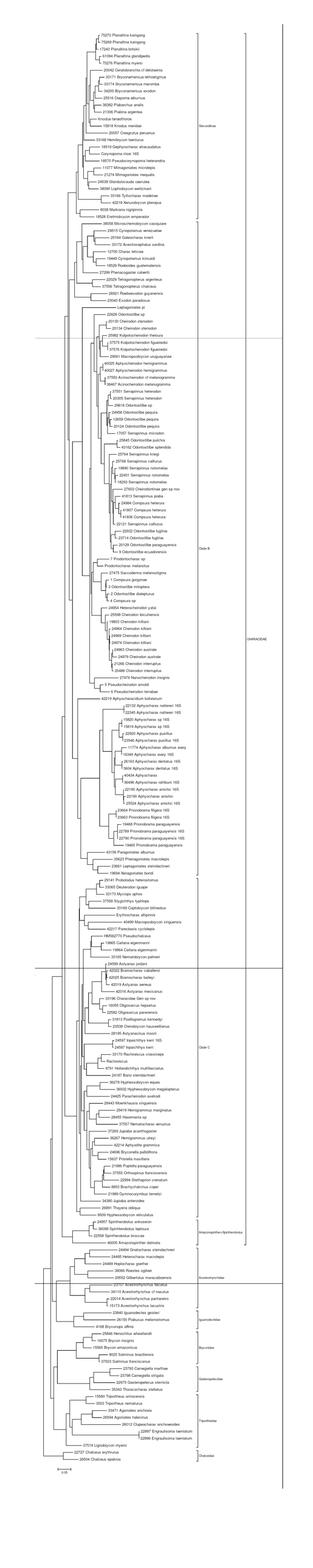

Supplement: S1 Fig — Best maximum likelihood tree showing the relationships among Triportheidae, Gasteropelecidae, Bryconidae, Acestrorhynchidae, Iguanodectidae, and Characidae. Chalceidae was used as outgroup. (PNG) [file pone.0196291.s001.png]

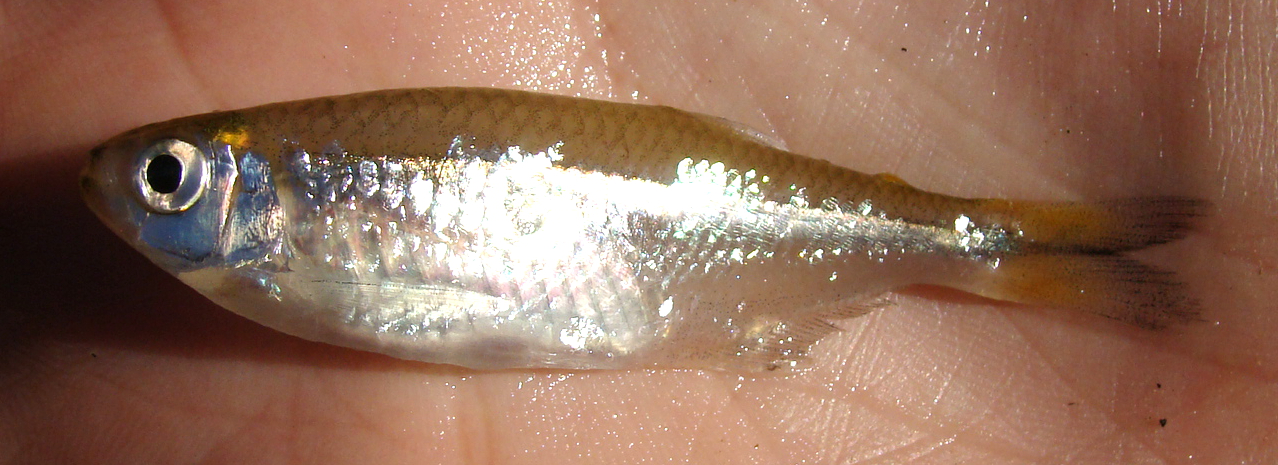

Supplement: S2 Fig — Planaltina kaingang, new species, non-type specimen in life, NUP 16406, municipality of Cândido de Abreu, rio Maria Flora, rio Ivaí basin, upper rio Paraná. (TIFF) [file pone.0196291.s002.tiff]
