# Supplementary material for: Molecular phylogeny of Planaltina Böhlke (Characidae: Stevardiinae) and comments on the definition and geographic distribution of the genus, with description of a new species
Source: PLoS One. 2018 May 16;13(5):e0196291. doi: 10.1371/journal.pone.0196291 (PMC5955486; doi:10.1371/journal.pone.0196291)
Supplement: S1 File — (DOCX) [file pone.0196291.s003.docx]

Supplementary File 1 - Sequences of primers used in present study.

| **Gene** | **Primer name** | **Primer sequence (5’-3’)** | **Source** |
| --- | --- | --- | --- |
| 16S | 16Sa-L | ACGCCTGTTTATCAAAAACAT | [1] |
|  | 16Sb-H | CCGGTCTGAACTCAGATCACGT | [1] |
|  |  |  |  |
| Cytb | L14841 | AAATCAAAGCATAACACTGAAGATG | [2] |
|  | H15915 | CCAATTTGCATGGATGTCTTCTCGG | [3] |
|  | LNF | GACTTGAAAAACCAYCGTTGT | Present study |
|  | H08R2 | GCTTTGGGAGTTAGDGGTGGGAGTTAGAATC | Present study |
|  |  |  |  |
| Myh6 | F329 | CCGCMTGGATGATCTACAC | [4] |
| 1stPCR | A3R1 | ATTCTCACCACCATCCAGTTGAA | [4] |
| Myh6 | A3F2 | GGAGAATCARTCKGTGCTCATCA | [4] |
| 2ndPCR | A3R2 | CTCACCACCATCCAGTTGAACAT | [4] |
|  | R1242 | ACAGGATTGAGATGCTGTCCA | [4] |
|  | Myh6COF1 | GACTGTTAACACCAAGAGAGT | Present study |
|  | Myh6COF2 | GTTATCCAGTATTTTGCAAGTATTGC | Present study |
|  | Myh6COR1 | TTGAACATCTTCTCATACAC | Present study |
|  | Myh6COR2 | TTCTCATACACTGACTTAGCCAGTGC | Present study |
|  |  |  |  |
| RAG1 | 2510F | TGGCCATCCGGGTMAACAC | [5] |
| 1stPCR | 4090R | CTGAGTCCTTGTGAGCTTCCATRAAYTT | [5] |
| RAG1 | 2535F | AGCCAGTACCATAAGATGTA | [5] |
| 2ndPCR | 4078R | TGAGCCTCCATGAACTTCTGAAGRTAYTT | [5] |
|  | Rag1CF1 | ACCCTCCGTACTGCTGAGAA | Present study |
|  | Rag1CF2 | TACCGCTGAGAAGGAGCTTC | Present study |
|  | Rag1CF3 | GAGAAGGAGCTTCTCCCAGG | Present study |
|  | Rag1CF4 | GCTTCCATCAGTTTGAGTGG | Present study |
|  | Rag1CF5 | CAGCTCTTGGAACATAGGCATCA | Present study |
|  | Rag1CR1 | CGTCGGAAGAGCTTGTTGCC | Present study |
|  | Rag1CR2 | TGTTGCCAGACTCATTGCCCTC | Present study |
|  | Rag1CR3 | CCCTCGCTGGCCCAGGCACC | Present study |
|  | Rag1CR4 | ATCTCGTTCCACAATCTCAGGC | Present study |
|  | Rag1CR5 | CATGGGCCAGTGTCTTGTGGAGGT | Present study |
|  |  |  |  |
| RAG2 | 164F | AGCTCAAGCTGCGYGCCAT | Present study |
| 1stPCR | RAG2-R6 | TGRTCCARGCAGAAGTACTTG | [6] |
| RAG2 | 176R | GYGCCATCTCATTCTCCAACA | Present study |
| 2ndPCR | Rag2Ri | AGAACAAAAGATCATTGCTGGTCGGG | Present study |

References

1. Palumbi SR. Nucleic acids II: The polymerase chain reaction. In: Hillis D, Moritz C, Mable B (editors). *Molecular Systematics*. Sinauer Associates Inc.: Massachusetts; 1996. pp. 205–247.

2. Kocher TD, Thomas WK, Meyer A, Edwards SV, Pääbo S, Villablanca FX, Wilson A. Dynamics of mitochondrial DNA evolution in animals: amplification and sequencing with conserved primers. Proc Natl Acad Sci. 1989; 86: 6196–6200.

3. Irwing DM, Kocher TD, Wilson AC. Evolution of the cytochrome *b* gene of mammals. J Mol Evol. 1991; 32: 128–144.

4. Li C, Ortí G, Zhang G, Lu G. A practical approach to phylogenomics: The phylogeny of ray-finned fish (Actinopterygii) as a case study. BMC Evol Biol. 2007; **7**: 44.

5. Li C, Ortí G. Molecular phylogeny of Clupeiformes (Actinopterygii) inferred from nuclear and mitochondrial DNA sequences. Mol Phylogenet Evol. 2007; 44: 386–398.

6. Lovejoy NR, Collette BB. Phylogenetic relationships of new world needlefishes (Teleostei: Belonidae) and the biogeography of transitions between marine and freshwater habitats. Copeia. 2001: 1: 324–338.
